# LeafGo: Leaf to Genome, a quick workflow to produce high-quality de novo plant genomes using long-read sequencing technology

**DOI:** 10.1186/s13059-021-02475-z

**Published:** 2021-09-03

**Authors:** Patrick Driguez, Salim Bougouffa, Karen Carty, Alexander Putra, Kamel Jabbari, Muppala Reddy, Richard Soppe, Ming Sin Cheung, Yoshinori Fukasawa, Luca Ermini

**Affiliations:** Core Labs, King Abdullah University of Science and Technology (KAUST), Thuwal, Makkah, 23955-6900 Saudi Arabia

**Keywords:** Long-read sequencing, Chromosome-level draft genome, High molecular weight DNA extraction, *Eucalyptus*, Peanut, *Arachis*, Genomic standardized workflow

## Abstract

Currently, different sequencing platforms are used to generate plant genomes and no workflow has been properly developed to optimize time, cost, and assembly quality. We present LeafGo, a complete de novo plant genome workflow, that starts from tissue and produces genomes with modest laboratory and bioinformatic resources in approximately 7 days and using one long-read sequencing technology. LeafGo is optimized with ten different plant species, three of which are used to generate high-quality chromosome-level assemblies without any scaffolding technologies. Finally, we report the diploid genomes of *Eucalyptus rudis* and *E. camaldulensis* and the allotetraploid genome of *Arachis hypogaea*.

**Supplementary Information:**

The online version contains supplementary material available at 10.1186/s13059-021-02475-z.

## Background

Plants represent the dominant kingdom of life in terms of Earth biomass [1], and through colonization of terrestrial and aquatic habitats, are responsible for maintaining ecological and atmospheric balance. Despite being globally distributed, climate change and anthropogenic activities are massively impacting current plant diversity with repercussions for ecophysiology, distribution, and interactions with other organisms [2, 3]. Sequencing present-day plant genomes to better understand genomic diversity is an important requirement for gauging plants’ susceptibility to climate change.

In the last decade, rapid advances in short-read sequencing technology have resulted in the availability of over 300 plant species genomes, of differing quality [4]. Recently, long-read sequencing methods (Pacific Biosciences, PacBio, and Oxford Nanopore Technology, ONT) are becoming more accessible while technological advances have led to increases in the base accuracy and the sequencing length, as well as a significant reduction in cost per base of sequence [5]. The main benefit of long-read sequencing technologies for genomics, compared to the more dominant short-read/Illumina sequencing, is the ability to assemble genomes relatively easily by linking reads that span across repetitive genomic regions. This property when combined with ultra-long reads, highly accurate sequencing, and complementary scaffolding technologies has thereby enabled highly accurate telomere to telomere assemblies [6–8]. The many benefits of long-read sequencing have driven demand for high-quality high molecular weight (HMW) DNA, and led to advances in sequencing technologies and genome assembly tools.

In the case of plants, genomes can be large and highly repetitive, making long-read sequencing ideal for genome assembly [9–11]. However, it is often difficult to extract HMW DNA suitable for long-read sequencing from plants [12–14]. Plants have tough cell walls and contain high levels of metabolic contaminants, such as polyphenols and polysaccharides [15–17] which are difficult to eliminate and impact sequencing quality. Furthermore, a plethora of new initiatives have emerged to sequence millions of species, including those from the plant kingdom [18, 19].

**Fig. 1:**
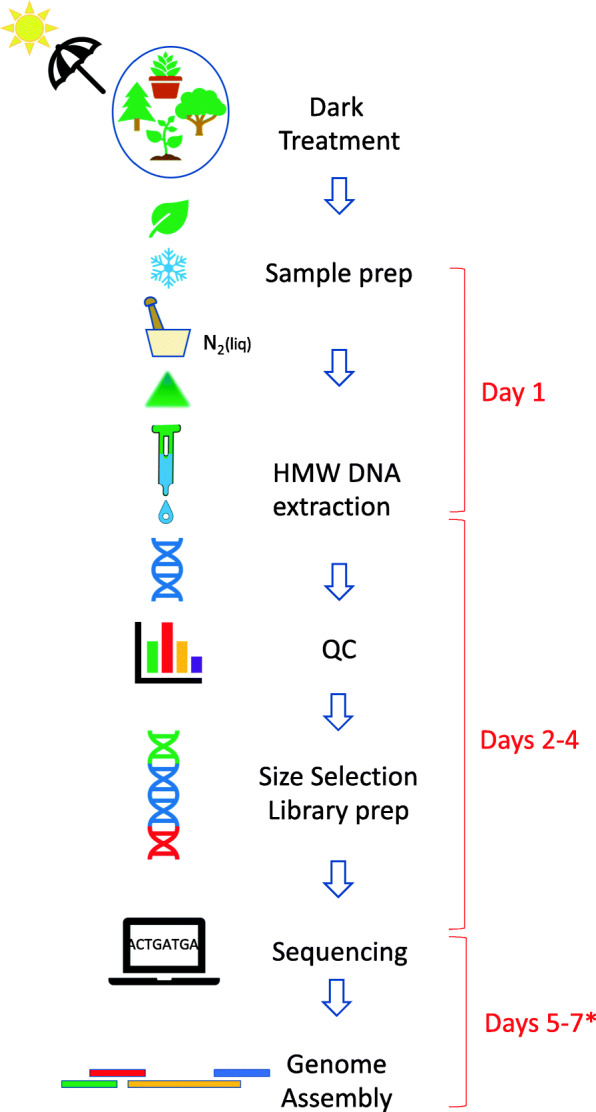
Plant long-read sequencing workflow. Asterisk indicates sequencing time depends on genome size and ploidy. Seven days completion time is based on a diploid organism with a haploid genome < 0.6–1 gigabases

To address the need to sequence new plant genomes, and to achieve the best operating conditions for accomplishing high-quality de novo genomes with one technology, we present and discuss the development of LeafGo. This is a complete workflow designed to generate de novo genomes from plant tissue with relatively modest resources within 7 days for genomes less than approximately one gigabase (Gb) and increasing incrementally for larger or more complex genomes (Fig. 1).

To develop LeafGo, we selected ten plant species (*Arachis hypogaea* subsp. *fastigiata, Brassica rapa* subsp*. chinensis, Distichlis palmeri*, *Eucalyptus rudis* subsp. *rudis*, *E. camaldulensis* subsp*. obtusa, Pennisetum glaucum, Salicornia bigelovii, Salvadora persica, Solanum melongena,* and *Zea mays*; Additional file 1: Table S1) from seven diverse plant taxonomic families for HMW DNA extraction and long-read sequencing. Maize (*Z. mays*), pearl millet (*P. glaucum*), and peanut (*A. hypogaea*) are well-studied, globally important crops responsible for feeding millions of people with large, highly repetitive genomes [10, 11, 20–25]. Similarly, bok choy (*B. rapa*) and eggplant (*S. melongena*) are high production crops important for human nutrition with published genomes [26–28]. Nipa grass (*D. palmeri*), dwarf saltwort (*S. bigelovii*), and toothbrush tree (*S. persica*) are lesser-researched plants without published genomes that are, or could be developed into, agriculturally/pharmacologically important crops [29–32]. Finally, the flooded gum (*E. rudis*) and river red gum (*E. camaldulensis*) trees were selected. Eucalypts are the most commonly planted hardwood trees in the world due to their fast growth, environmental adaptability, and many commercial uses [33]. However, good-quality HMW DNA is relatively difficult to extract from eucalypts due to their high phenolic and polysaccharide content [12, 17, 34]. Moreover, of the > 800 eucalypt species, only a few high-quality genomes have been published [33, 35, 36].

This paper provides the rationale for the adoption of LeafGo, a workflow that combines different resources to generate de novo genomes of different plants. LeafGo consists of a robust HMW DNA extraction method, library preparation and sequencing approach, and genome assembly suggestions. This work also provides the community with three high-quality de novo genomes of *E. camaldulensis*, *E. rudis,* and *A. hypogaea*.

## Results

One of the most challenging aspects of long-read sequencing is applying stringent quality controls at every step of the laboratory workflow in order to obtain good-quality sequencing results. We outline the best conditions to extract HMW DNA and to process it into long-read sequencing libraries for the PacBio and ONT platforms. In particular, we prepared PacBio continuous long read (CLR) and high fidelity (HiFi, also known as circular consensus sequencing, CCS) libraries and sequenced them on the latest PacBio platforms, Sequel I and Sequel II. For the *Eucalyptus* species, libraries were produced and sequenced with the GridION platform demonstrating the suitability of the laboratory component of LeafGo for ONT sequencing [34, 37, 38] (see Additional file 1: Oxford Nanopore Technology Sequencing; Tables S2 and S3; Fig. S1). Finally, we compared CLR and HiFi data using the latest tools for genome assembly and assembled the two *Eucalyptus* species and *A. hypogaea* into high-quality draft genomes.

### DNA extraction, quality controls, library, and long-read sequencing

#### DNA extraction

**Fig. 2:**
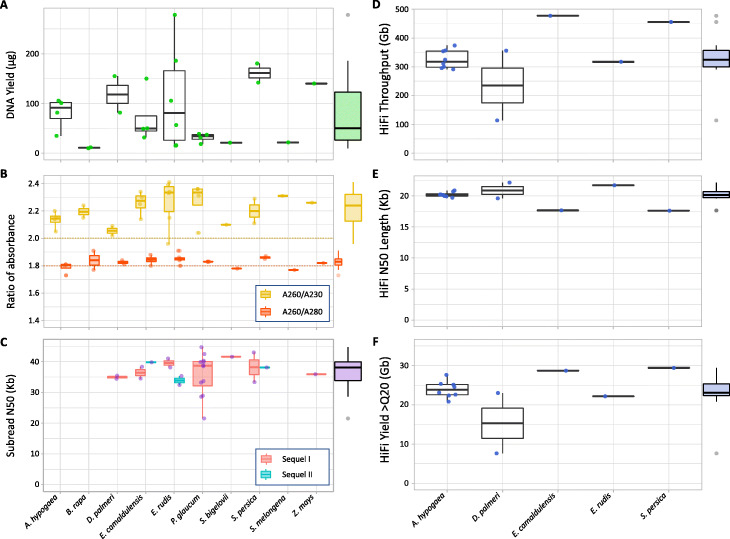
DNA extraction and long-read sequencing output from study plant species. Yield (**A**) and absorbance ratios (**B**) of extracted HMW DNA from ten study plant species. Subread N50 of CLR libraries on Sequel I and Sequel II for seven of the ten study plant species (**C**). The total throughput (**D**), subread N50 length (**E**), and Q20 yield (**F**) for HiFi libraries sequenced on Sequel II for five study plant species. The average for all data is plotted along the margin. CLR sequencing was not completed for *A. hypogaea*, *B. rapa,* and *S. melongena*

The extraction protocol implemented in LeafGo generated large amounts of HMW DNA with high purity within a day and using minimal resources and effort. The protocol yielded high-quality HMW DNA in 27 separate extractions from ten different plant species over different days by different technicians. The yield (per 1 g wet weight of leaf) ranged from 10 to 278 μg (average ± standard deviation [SD], 79.6 ± 71.6) with high variability in the *Eucalyptus* species, compared to the other species (Fig. 2A). All the extracted HMW DNA had high purity and integrity, despite the different composition, including potential contaminants, between species [12, 29, 32, 34, 36]. Notably, the *Eucalyptus* samples showed signs of oxidation during lysis, such as dark coloration of the solution; however, the quality of DNA was not compromised, without the need for added antioxidants [12, 34]. The absorbance ratios indicated low levels of contaminants, such as protein, carbohydrates and phenolics, and high purity (average *A*_260/280_ = 1.83 ± 0.05 SD and *A*_260/230_ = 2.21 ± 0.13 SD; Fig. 2B) [9, 34, 36, 39]. The integrity of the extracted genomic DNA (gDNA) was assessed by pulsed-field gel electrophoresis and capillary electrophoresis. The extracted samples typically showed a DNA smear ranging from approximately > 15 to < 150 Kb and a mode of over 80 Kb for all samples (Additional file 1: Fig. S2 and S3). Our results are comparable to, or improve on, similar studies in terms of DNA yield, fragment length, and purity but with a simpler and less toxic extraction protocol [12, 17, 34] that produces HMW gDNA suitable for long-read sequencing in 1 day.

#### PacBio CLR and HiFi sequencing

The extracted HMW gDNA from eight of the species (*Arachis hypogaea, Distichlis palmeri, Eucalyptus rudis, E. camaldulensis, Pennisetum glaucum, Salicornia bigelovii, Salvadora persica,* and *Zea mays*), was processed into 26 CLR and 7 HiFi libraries prior to sequencing with the PacBio Sequel I (22: 1M SMRT cells) and Sequel II (17: 8M SMRT cells). SMRTLink analysis (Additional file 1: Table S4) showed that sequence statistics for both CLR and HiFi libraries were above the PacBio recommended specifications [40] with optimal internal controls metrics indicating that no inhibition of the sequencing reaction was observed.

The CLR libraries (Additional file 1: Fig. S4A to S4D) showed a mode > 30 kb with few shorter fragments. The sequencing results confirmed that the extracted gDNA was of good purity and of high molecular weight. The average CLR throughput per Sequel I SMRT cell was 11.5 Gb (± 4.7 Gb [SD]; 3.3–18.1 Gb [min–max]) while the Sequel II yielded 167.6 Gb/SMRT cell (± 26.1 Gb; 140.8–195.4 Gb) (Additional file 1: Fig. S4E and S4F). The average N50 subread length was 36.6 kilobases (Kb) (± 5.5 Kb; 21.5–44.7 Kb) for the 22 Sequel I CLR libraries, and 36.4 Kb (± 3.2 Kb; 32.4–39.8 Kb) for the four Sequel II CLR libraries (Fig. 2C). Sheared DNA was tested for CLR library preparation; however, there was no obvious improvement in yield or N50, as previously shown with ONT libraries [34], and unsheared DNA was used for all other libraries. To test whether library loading affected the subread N50 length, some SMRT cells were intentionally underloaded. There was a borderline significant correlation (Additional file 1: Table S5 and Fig. S5) between ZMW (zero mode waveguide) occupancy or library underloading (high P0%) and subread N50 (Spearman’s rho = 0.42, *p* = 0.0499) suggesting a small benefit in subread N50 at the expense of throughput yield; unsurprisingly, library loading (determined from P0% and P1%) was significant correlated with throughput yield (Spearman’s rho = − 0.84 and 0.86, *p*-value < 0.00005). Although not presented here, initial testing with higher size-selected CLR libraries (35 Kb and 40 Kb) yielded diminished sequencing results with inconsistent throughput and lower N50 sizes. For this reason, we exclusively used 30-Kb size-selected CLR libraries. The reason for the suboptimal results with higher size-selected libraries is not clear and might require follow up studies.

The HiFi libraries showed a mode of approximately 20 Kb (Additional file 1: Fig. S6) and, when sequenced with Sequel II, yielded an average total throughput of 322.9 Gb/SMRT cell (± 87.6 Gb; 113.9–477.3 Gb), with the Q20 yield of 23.3 Gb (± 5.4 Gb; 7.6–29.4 Gb) and subread N50 of 20.1 Kb (± 1.3 Kb; 17.6–22.2 Kb) as shown in Fig. 2D–F. The total throughput and Q20 yield were high for all the samples except for one *D. palmeri* HiFi library that was underloaded (P1% = 13%); subsequent resequencing of the library with optimal loading yielded more typical results (356.3 Gb total throughput; 23 Gb Q20 yield).

### De novo genome assembly of *Eucalyptus* and *Arachis* species

Assembling a de novo genome requires a combination of coverage, read length, base quality, and computational resources. An accurate reconstruction, preferably in a short time frame, is indeed crucial, as both the continuity and base accuracy of an assembly can affect the quality of the genome. LeafGo bioinformatics recommendations are based on testing existing and recently developed tools to optimize both genome quality and computational time, a commonly limited resource. To evaluate the best approach, both HiFi and CLR sequencing data of two diploid eucalypts (2n = 2x = 20) and HiFi data for the allotetraploid peanut (2n = 4x = 40) were assessed and assembled.

#### Quality control of raw sequencing data

The first bioinformatics step is the quality control (QC) of the raw sequencing data. Platform-specific metrics represent the first informative statistics for the overall quality of the sequencing run (Additional file 1: Table S4). However, a more comprehensive QC step was performed using LongQC [41]. LongQC, or similar software, allows rapid and in depth cross-platform QC of the raw sequencing data. LongQC results for the *Eucalyptus* species and *A. hypogaea* sequencing data are shown in Additional file 1: Fig. S7 and S8.

All HiFi data for the three species are of good quality as indicated by the high scores for per read base calling accuracy (Additional file 1: Fig. S7A, S7F and S7M) and by a normal distribution of per read coverage, except for the slight bimodality in *E. rudis* (Additional file 1: Fig. S7C, S7H and S7O). Both *Eucalyptus* species showed a similar GC content and sequence complexity (Additional file 1: Fig. S7B, S7G and S7D, S7I). The GC content for all the *Eucalyptus* reads shows a sharp unimodal distribution around a mean of 0.39 (± 0.03 [SD]), but with an upper sub-mode outlier near 0.55. Closer inspection of this higher GC content peak revealed the presence of telomere repeats.

The GC content of *Arachis* (Additional file 1: Fig. S7N) presents a sharp unimodal distribution around a mean of 0.36 (± 0.05 [SD]), but two sub-modes are observed: an upper sub-mode outlier near 0.55 like the eucalypts, and one lower sub-mode outlier near 0.15. A closer investigation of the lower sub-mode showed the presence of centromere repeats [42]. No artificial sequence adapters are present in the flanking region for either datasets (Additional file 1: Fig. S7E, S7L and S7Q).

In contrast, CLR data for the two eucalypts does not achieve the same level of quality shown by HiFi data. The Phred scores are not provided in CLR mode and thus read base calling accuracy cannot be directly assessed. The sequence complexity of CLR is lower (sequences with low complexity, 0–40% and 0–20% in CLR and HiFi, respectively), and the flanking regions seem to extend over a hundred bases pointing to the possibility of artificial sequences (Additional file 1: Fig. S8B, S8F and S8D, S8H). Both *Eucalyptus* species’ CLR sequences have a similar GC content to that for HiFi data. The GC content for all the reads has a sharp normal distribution around a mean of 40% (± 4%), but the upper sub-mode peak, indicative of telomere repeats and present in HiFi data, is not found within the CLR distribution. Surprisingly, CLR sequencing may be less sensitive at detecting telomere repeats than HiFi.

#### Genome assemblies

Prior to assembly, *k*-mer counting can provide insights into the genome size of an unknown genome. A *k*-mer assessment on HiFi data using GenomeScope 2.0 [36] estimated the genome size of *E. rudis*, *E. camaldulensis,* and *A. hypogaea* as 506 Mb, 510 Mb, and 2.54 Gb, respectively (Additional file 1: Fig. S9). The heterozygosity level was also estimated using the same *k*-mer approach and was relatively high in both eucalypts (ab: *E. camaldulensis*, 2.19%; *E. rudis*, 1.57%) which is expected to affect genome assembly [37] and estimated genome size [38]. The patterns of nucleotide heterozygosity rates shown by *A. hypogaea* follow the expected distinct patterns for allotetraploid genomes [43] (Additional file 1: Fig. S9C).

The three genomes were assembled with tools optimized for long-read sequences: Canu [44] for CLR data (only the two eucalypts) and hifiasm [45] for HiFi data. A comparison among different assemblers has shown Canu is an efficient assembler for CLR PacBio data [46]. For HiFi generated data, we carried out a comparison among different assemblers (hifiasm v0.8 [45], HiCanu v2 [44], Flye v2.8.1 [47], Wtdbg2 v2.5 [48]) and found that hifiasm outperformed the other assemblers (Additional file 1: Tables S6 and S7).

##### Eucalyptus diploid genomes

The overall genome assembly statistics are shown in Table 1. For the two eucalypts, the assembled genome size based on the HiFi and on the CLR data is similar for *E. rudis* and *E. camaldulensis*. The HiFi assembly is superior to the CLR assembly for both *Eucalyptus* species, which is in agreement with HiFi-based assemblies for other species [8, 44, 49]. The contig N50/N90 and L50/L90 show noticeably higher contiguity in the HiFi assemblies compared to the CLR assemblies. Furthermore, the HiFi assemblies consistently produced the longest contigs (*E. rudis,* 61.8 Mb, 33.7 Mb; *E. camaldulensis,* 69.1 Mb, 58.1 Mb; for HiFi and CLR, respectively).

**Table 1:** Genome assembly statistics for two *Eucalyptus* species and *A. hypogaea*. We calculated the assembly statistics using Quast. CLR-based assemblies were 3-cycle polished as detailed in “Methods”. Results are based on the purged assemblies (see “Methods”).

Plants	Type	Size^d^ (Mb), ≥ 1 Mb|total	No. contigs^d^, ≥ 1 Mb|total	N50 (Mb)/L50	N90 (Mb)/L90	Longest contig (Mb)	Alternative size (Mb)
*E. rudis*^a^	HiFi (~ 40×)	531|549	26|331	36.0/7	7.3/15	61.8	425
	CLR (~ 50×)	506|518	44|138	16.3/11	5.2/30	33.7	399
*E. camaldulensis*^b^	HiFi (~ 51×)	525|532	14|149	41.4/5	23.2/12	69.1	520
	CLR (~ 230×)	516|523	28|77	29.3/7	8.5/19	58.1	570
*A. hypogaea*^c^	HiFi (~ 74×)	2,564|2,623	114|1417	42.3/22	10.37/69	90.3	51

^a^*E. rudis* unknown genome size. Coverage estimated based on assembly size

^b^*E. camaldulensis* reference genome size, 558.6 Mb (AC: GCA_014182705.1)

^c^*A. hypogaea* reference genome size, 2557 Mb (AC: GCF_003086295.2)

*N50* the smallest length contig at which the cumulative contig lengths equal to 50% of the assembled size, *L50* N50 contig count, *N90* the smallest length contig at which the cumulative contig lengths equal to 90% of the assembled size, *L90* N90 contig count

^d^Metric calculated based on (1) minimum contig length cut-off of 1Mb or (2) no cut-off

**Fig. 3:**
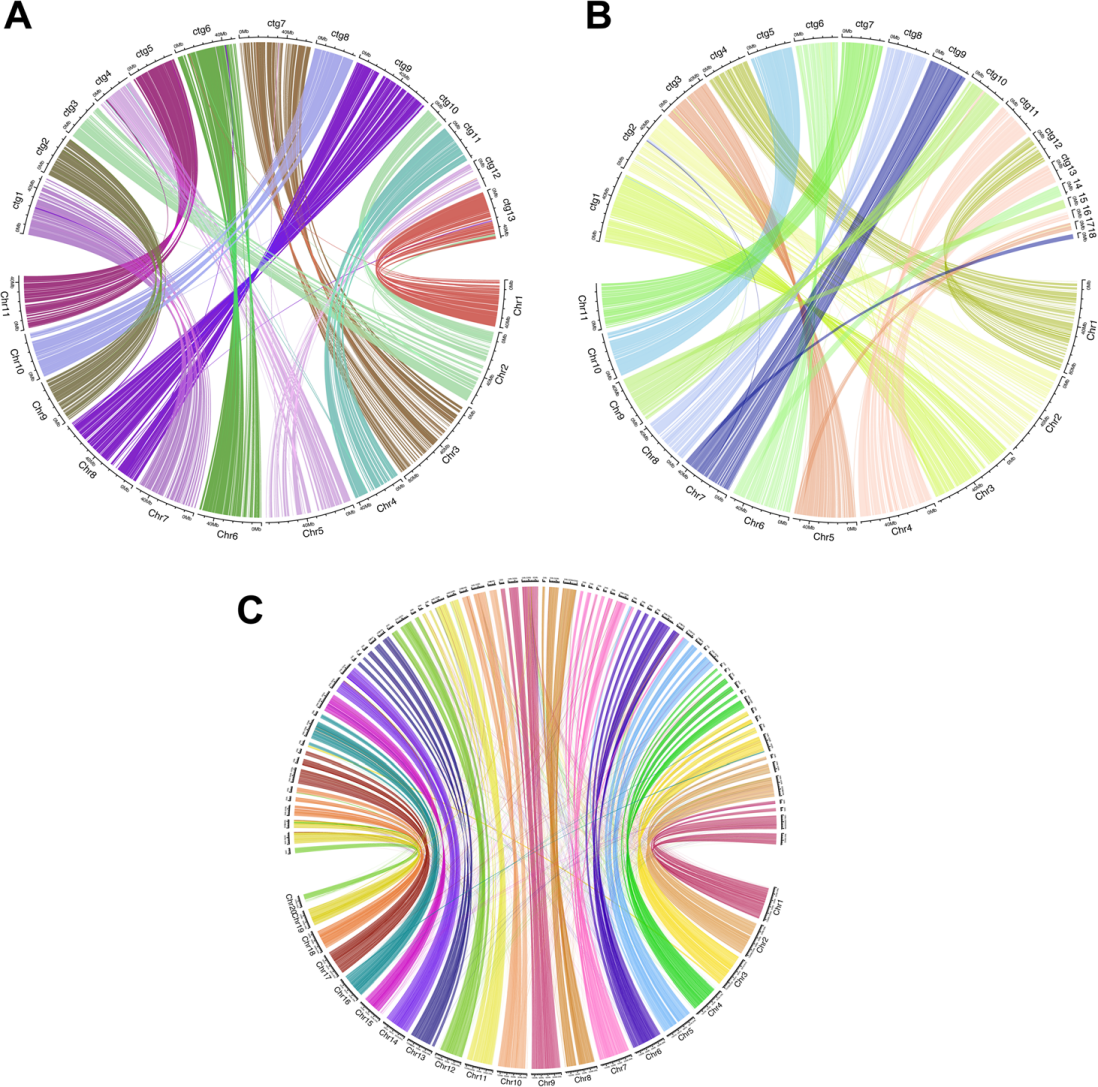
Chord diagrams of *E. camaldulensi*s, *E. rudis, and A. hypogaea* de novo assemblies mapped against a reference genome. Alignment of *E. camaldulensis* (**A**), *E. rudis* (**B**), and *A. hypogaea* (**C**) HiFi assemblies against *E. grandis* (**A** and **B**) and *A. hypogaea* (**C**) reference genomes

The assembly haploid genome sizes were estimated by separating haplotypes and purging haplotigs, and the completeness was assessed using BUSCO scores (Additional file 1: Table S8). Based on the primary haplo-purged assembly, the haploid genome size of *E. rudis* varies between 518 Mb (CLR) and 549 Mb (HiFi) and produced an estimated ploidy of 1.77N. The high complete BUSCO scores (> 96%) for the primary contigs and a relatively high score for the alternative contigs (CLR, 73.4%; HiFi, 87.2%) indicate a reasonable separation of the haplotypes. In addition, the alternative contig set for the CLR assembly shows a lower degree of completeness which further demonstrates the superiority of the HiFi assembly. The haploid genome size for *E. camaldulensis* is about 523 and 532 Mb based respectively on CLR and HiFi primary assemblies. Both the primary and the alternative contig sets show high BUSCO scores (primary, > 97%; alternative, > 93%) in both CLR and HiFi assemblies. The estimated ploidy of the assembly after haplo-purging is over 1.98 N which, taken together with the BUSCO scores, suggests that the assembly is comprehensive. Alignment to the *E. grandis* genome [33] shows that our HiFi *E. camaldulensis* assembly has 9 full chromosomes with the remaining two chromosomes spanned almost fully by two contigs each (Fig. 3). Similarly, the *E. rudis* HiFi assembly has five full chromosomes and the remaining chromosomes spanned almost fully by two to three contigs each. In terms of computational time, assembling the draft genome with HiFi data is 65 times quicker than using CLR for similar coverage (40× HiFi, 53 CPU hrs; 50× CLR, 3444 CPU hrs; *E. rudis*). This gap surges to 895 times when the coverage of CLR over HiFi increases about 4 times (51× HiFi, 81 CPU hrs; 230× CLR, 72491 CPU hrs; *E. camaldulensis*). The two *Eucalyptus* genomes show the same assembly complexity and similar computational time is expected. The two *Eucalyptus* species were further identified by phenotypic and *in silico* inspection [50, 51] (see Additional file 1: Phenotypic and *in silico* identification of the *Eucalyptus* species; Fig. S10).

The recent assembly of *E. pauciflora*, a genome of similar size and complexity, offers a possible comparison with our assemblies. A hybrid assembly strategy, with ONT long-read scaffolding contigs generated by Illumina short reads, has been adopted to generate the *E. pauciflora* genome [36]. The LeafGo approach is simpler and less labor intensive by using only one long-read technology, and produced genomes with twelve times more contiguity (contig N50 36/41 Mb vs 3.2 Mb, *E. rudis/camaldulensis, E. pauciflora* respectively) with a hundred times less computational resources (see Additional file 1: Genome assembly: computational resources).

##### Arachis hypogaea allotetraploid genome

The assembly of the allotetraploid genome of *A. hypogaea* was carried out only with HiFi data. This resulted in a 2623-Mb genome size with 42.3 Mb and 10.37 Mb contigs N50 and N90 respectively. Notably, the *A. hypogaea* genome is a large genome rich in repetitive content [25] and a large quantity of long reads are needed to help bridge difficult regions in the genome. Our assembly proves to be more contiguous than a previously reported assembly generated only by older PacBio technology showing a lower N50 of 1.5 Mb and N90 of 0.34 Mb [25]. Alignment to the *A. hypogaea* reference genome (GCA_003086295.2) shows that our HiFi *A. hypogaea* assembly is comprehensive where the majority of the 20 chromosomes are spanned by a few contigs each. A closer inspection of the alignment revealed central and peripheral chromosomal regions (likely telomeres and/or centromeres) not fully assembling into the larger contigs and therefore falling short of chromosome-level assembly (Fig. 3C). The assembly, indeed, faces the challenge of the repetitive nature of the peanut genome [24], richer in repetitive content than the eucalypt ones (*E. rudis*, 37.4%; *E. camaldulensis*, 38.3%; *A. hypogaea*, 82.9%; Additional file 1: Fig. S9). The high BUSCO score of 97.5% supports the high quality and completeness of our HiFi assembly, although the computational time to assemble the large peanut allotetraploid genome with HiFi data is longer compared to the smaller diploid eucalypt genomes (74× HiFi, 1081 CPU hrs, *A. hypogaea*).

##### Time estimates

The estimated total time from raw reads to HiFi/CCS reads to the assembly of a high-quality contiguous draft for a diploid genome of 0.6 to 1.0 Gb is less than one day which increases to approximately 2 days for a tetraploid genome of 2.5 Gb (see Additional file 1: Genome assembly: computational resources). When combined with time estimates of HMW DNA extraction (1 day), HiFi library preparation and sequencing (5 days) and assembly, a high-quality draft genome of 0.6–1.0 Gb can be prepared from plant samples within a minimum of 7 days, depending on available compute resources. This time increases for a polyploid (2.5 Gb) genome in which sequencing will require approximately 9 additional days bringing the time up to 16 days (Additional file 1: Fig. S11). All time estimates are based on sequencing using a single Sequel II machine and will vary according to coverage requirements.

## Discussion

The current era of genomics is very promising with ambitious projects to sequence much of life on Earth [18, 19, 52]. The ideal goal for these projects would be to produce high-quality error-free, gapless or near-gapless haplotype-resolved genome assemblies. Very few gapless chromosome-level reference genomes today exist and typically require two or more different technologies like PacBio/ONT long reads, Illumina short and 10X linked reads sequencing, optical mapping, and Hi-C [5, 9, 11, 53]. In particular, plants prove to have some of the most challenging genomes to assemble as they are rich in repetitive content, with high levels of heterozygosity and complex polyploidy [54]. Assembling a high-quality plant genome with complete or near complete chromosomes therefore requires high coverage, long-read length, and high-quality sequences with a low error rate. Recently, PacBio has improved the circular consensus sequencing approach to generate long (≥ 15 Kb) HiFi reads with base accuracy upwards of 99.8% [8], and we took advantage of this progress and developed LeafGo, a streamlined workflow able to produce a high-quality draft plant genome from plant tissue within 7 days.

LeafGo is designed to be used as a rapid one-pass approach for assembling a high-quality draft genome that generates data suitable for most plant species. Our workflow targets a crucial problem: optimally assembling high-quality de novo genomes using only one technology. Our goal was achieved by considering the overall procedure, and modifying and optimizing each single step for consistent results. On this basis, we provide a robust HMW DNA extraction method, guidance for library preparation and sequencing, and recommendations for genome assembly.

The use of one sequencing technology such as PacBio is a distinctive advantage of LeafGo, although compatibility of the extracted HMW DNA with long-read ONT sequencing is also demonstrated. The adoption of other sequencing technologies might improve the contiguity of a genome assembly, but a significant investment of additional resources may be needed, such as ultra-high molecular weight DNA extractions, increased wet-lab labor and time, additional equipment and reagents, various software packages, and extensive bioinformatics analysis with manual intervention [5, 9, 11, 52, 53].

LeafGo uses a modified column-based gDNA extraction protocol delivering high molecular weight DNA of excellent quality. All the extracted gDNA samples had a mode length of over 80 Kb, with optical density ratios indicating absence of organic contamination. We then benchmarked the effectiveness and utility of two different methods of PacBio library preparation and sequencing, HiFi, and CLR. The HiFi protocol proved to generate high base-level accuracy long reads, while the CLR protocol produced subreads of considerable length. The CLR subread N50 is further evidence of the quality of the extracted HMW DNA. The averaged CLR N50 subread length was greater than 36 Kb on both Sequel I and Sequel II platforms, exceeding therefore the N50 of many recent studies [11, 49, 52, 55], and we introduced the N50 subread length parameter as a quality control for assessing HMW DNA extractions. It should be highlighted that although we extracted high-quality HMW DNA from ten different species, our extraction method might not work for all plant species.

Although the eucalypt genomes assembled in this manuscript with both CLR and HiFi data are very contiguous, the higher base-level accuracy given by HiFi improves the assembly considerably thus removing the need for polishing with short-read sequencing [44]. Our results emphasize several strengths of the HiFi technology. Compared to the CLR assemblies, HiFi assemblies demanded less computational requirements, had higher BUSCO scores, showed several fold improvement of contig N50/N90 and L50/L90, and generated more complete genome assemblies, as has been previously published [8, 45, 49]. In fact, HiFi sequencing data assembled with hifiasm produced near-chromosome-level haploid draft genomes.

The two *Eucalyptus* species genomes assembled with the LeafGo workflow will improve our genomic knowledge of eucalypts, which at the moment is relatively sparse, and will assist with conservation issues and commercial uses. There are more than 800 eucalypt species, but only three genomes have been published: *E. grandis* [33], *E. pauciflora* [36], and *E. camaldulensis* [35]. The high-quality draft genome of *E. camaldulensis* generated with LeafGo improves upon the published and highly fragmented genome produced by short-read sequencing [35], and the unpublished reference-based and less contiguous assembly recently produced from ONT long-reads [56] as shown by the assembly metrics (LeafGo de novo assembly contig N50 of 41.4 Mb; ONT reference-based assembly contig N50 of 2.5 Mb). The first genome assembly of *E. rudis* is generated of mostly complete chromosomes and shows a primary haploid genome size of 549 Mb (HiFi assembly) which is similar to other eucalypts [33, 35, 36, 56].

Polyploidy plays an important role in plant evolution affecting phenotypic diversification, ecological tolerance, and species richness. The *A. hypogaea* genome is a large polyploid genome, about four times larger than the above eucalypt species. It carries two sets of chromosome pairs, originating from a hybridization event of two distinct ancestral *Arachis* species 9400 years ago (*A. ipaensis*, genome A; *A. duranensis*, genome B) which generated the allotetraploid genome of *A. hypogaea* (AABB-type genome; 2n = 4× = 40 chromosomes; genome size of ~ 2.5 Gb) [57].

Recently, different allotetraploid plant genomes were assembled with PacBio and other sequencing technologies [58]; here, we provide a highly contiguous and complete *Arachis hypogaea* genome assembly using only PacBio sequencing. Our assembly is comparable to the already published peanut genome [24, 25] and other polyploid plant species based on HiFi reads [45] consolidating the strength of our workflow. A careful examination of the alignment of our assembly against the published reference reveals that the source of fragmentation can be directly attributed to the central and peripheral regions (possibly centromeric and/or telomeric). Considering the high repeat content in the peanut genome (~ 82.9%), the contiguity of our assembly is impressive. Presently, for large and repetitive allotetraploid genomes such as the peanut, a scaffolding technology can improve the contiguity of the assembly [24] but it will take more time and add further costs. The generation of the *A. hypogaea* genome took 9 days longer than for a eucalypt species, due to the necessity of sequencing the larger genome at sufficient coverage with a consequential increase in platform sequencing time; however, significant reductions in sequencing time could be achieved by parallel sequencing on multiple machines.

Our HiFi assemblies of two diploid *Eucalyptus* genomes and the allotetraploid genome of *Arachis hypogaea* are therefore comparable to single technology assemblies based on high coverage CLR and HiFi assemblies of homozygous maize strains and, more relevant, to heterozygous plant species based on HiFi reads [45].

## Conclusions

The global initiatives to sequence and assemble genomes for thousands of eukaryotic life forms, including plants, do not yet have a published standardized workflow [18, 19, 52, 59, 60]. Pursuing this goal, LeafGo is a valuable tool as it provides the foundation to produce high-quality genome assemblies within 7 days. The timeline and the resulting genome contiguity therefore might be further improved by future bioinformatics and sequencing technology developments. However, currently LeafGo will be an extremely valuable resource for those scientists aiming for a fast and cost-effective genome assembly workflow. We envisage that the simplified and efficient LeafGo will be useful for plant researchers as well as specialized genome sequencing centers.

## Methods

### Sample collection and HMW DNA extraction

We chose ten plants from seven taxonomic families that are economically relevant and represent typical species that may require high-quality draft genomes (Additional file 1: Table S1). Leaves were collected after at least 48 h of dark treatment. If possible, an individual plant was used for leaf collection and HMW DNA extraction, as was the case with the species used for genome assembly (*A. hypogaea, E. rudis,* and *E. camaldulensis*). To dark treat an entire plant or a branch from a large tree, the target was covered with light-opaque black plastic sheets with a few holes that allowed air flow. The leaves were sprayed with ethanol and wiped to remove contaminating organisms. Leaves were removed and weighed before flash-freezing in liquid nitrogen.

HMW DNA was extracted by modifying a Qiagen Genomic protocol (Qiagen, Hilden, Germany). Briefly, frozen leaves were ground to a fine powder in a mortar and pestle under liquid nitrogen and stored at − 80°C. For extraction, 1 g of ground leaf powder was resuspended in lysis buffer with 1 mg/ml of proteinase K (19133, Qiagen) and 190 μg/ml of RNase A (19101, Qiagen). The lysis solution was incubated at 50 °C for at least 3.5 h with gentle rocking. Following centrifugation for 15 min at room temperature at 3220×*g* the supernatant was purified with genomic tip columns according to the manufacturer’s recommendations. After elution from the column, the DNA was precipitated with 0.7 volumes of isopropanol and inverted slowly until the appearance of a floating DNA mass, or “jellyfish”. The DNA was hooked out with a plastic loop and washed in fresh 80% ethanol for 1 min three times. Repeated washing in ethanol, a major modification in the protocol, has been shown to improve DNA purity without a decrease in yield [13]. The DNA pellet was resuspended in EB buffer overnight at room temperature. The yield and quality of the DNA was assessed with a Broad Range dsDNA Qubit assay (Q32853, ThermoFisher, Waltham, MA, USA) and NanoDrop 8000 (ThermoFisher). In the event that absorbance ratios were not optimal [9, 61], potentially indicating the presence of contaminants, a repeat bead clean was performed. To visualize the DNA smear, pulse field gel electrophoresis (PFGE) was run with a 1% agarose TBE gel over 24 h (Initial switch 1 s, final switch 25 s, 6 volt/cm, 120° included angle, Chef III, Bio-Rad, Hercules, CA, USA) with MidRange PFG and Lambda PFG markers (N0342S, N0341S, NEB, Ipswich, MA, USA). Alternatively, HMW DNA was also imaged with a gDNA 165 Kb kit (FP-1002-0275) on the Femto Pulse system (Agilent, Santa Clara, CA, USA). A detailed procedure is deposited in protocols.io (dx.doi.org/10.17504/protocols.io.bafmibk6).

### PacBio and Oxford Nanopore sequencing

#### CLR library preparation sequencing with PacBio Sequel I and II

SMRTbell libraries were constructed with SMRTbell Express Template Prep Kit 2.0 (Pacific Biosciences, Menlo Park, CA, USA, 100-938-900). The input DNA, with a size distribution mode predominantly above 80 Kb, was processed for SMRTbell library construction without any shearing (except for one library; Additional file 1: Table S2). Initially, 15–20 μg of gDNA was cleaned-up and concentrated with 0.45x AMPure PB (Pacific Biosciences, 100-265-900), then 10 μg of gDNA was used in the first enzymatic reactions to remove single-strand overhangs followed by the DNA damage repair, end-repair/A-tailing reaction and finally, adapter ligation. The SMRTbell libraries were then purified with 0.45x AMPure PB beads before size selection on the BluePippin system (Sage Science, Beverley, MA, USA) with a 30-Kb cut-off using a 0.75% agarose cassette with U1 ladder. Following size selection, the libraries were given a final 1x AMPure PB bead clean-up and eluted in 10 μl of EB (Pacific Bioscience, 101-633-500). The concentration and size of SMRTbell were assessed with the Qubit dsDNA assay kit (Thermo Fisher Scientific, Waltham, MA, USA; Q32854), and the Genomic DNA 165 Kb Kit on the FEMTO Pulse, respectively. For Sequel I, SMRTbell libraries were prepared for sequencing by annealing to Sequencing Primer v4 and Polymerase 3.0 with Sequel Sequencing kit 3.0 (Pacific Biosciences, 101-597-900) and Sequel Binding and Internal Control Kit 3.0 (Pacific Biosciences, 101-626-600), and SMRT Cell 1M v3 LR (Pacific Biosciences, 101-531-001) according to SMRT link Sample Setup v.7.0 instructions and sequencing for 10 or 20 h without any pre-extension time. For Sequel II, SMRTbells were annealed with primer v4 and polymerase with Sequel II Binding Kit 2.0 and Internal Control Kit 1.0 (Pacific Biosciences,101-842-900), SMRT Cell 8M (Pacific Biosciences, 101-389-001), and according to SMRT link Sample Setup v.8.0 and sequence for 15 or 30 h without any pre-extension time.

#### HiFi library preparation and sequencing with PacBio Sequel II

Fifteen to 20 μg of gDNA was diluted in EB and was sheared using g-TUBE or Megaruptor 2. Samples were loaded into g-TUBEs (Covaris, 520079) and sheared with an Eppendorf 5424 (Eppendorf, Hamburg, Germany) for 2 min each spin. A repeat spin was implemented to make sure the entire gDNA had passed through the orifice. Alternatively, samples were sheared using the Megaruptor 2 (Diagenode, Denville, USA) with Long Hydropores (E07010002) and Hydrotubes (C30010018). For shearing, small scale test shears were performed to make sure that the mode of the fragments was in the 15–20-Kb size range (size checked with FEMTO Pulse). A minimum 10 μg of sheared and 0.45x AMPure purified gDNA was carried into SMRTbell construction by using Express Template Prep Kit 2.0 + Enzyme Clean Up (101-843-100).

Additional step of nuclease treatment of the HiFi library after the ligation step was done to remove any non-intact SMRTbell templates. Following nuclease treatment, the SMRTbell library was size-selected with 3.1x of diluted 35% v/v AMPure PB beads or the BluePippin system. The concentration and size of HiFi SMRTbell were assessed with Qubit dsDNA assay kit and gDNA 165 Kb kit of FEMTO Pulse, respectively. The SMRTbell libraries were annealed and bound with sequencing primer v2 (101-847-900), and Sequel II DNA polymerase 2.0 from Sequel II Binding kit 2.0, 101-842-900, respectively, using conditions specified in SMRT Link Sample Setup v.8.0. The final sample bound complex was sequenced with Sequel II Sequencing Kit 2.0 (101-820-200), and SMRT cell 8M Tray (101-389-001), and ran for 30 h with 2 or 4 h of pre-extension.

#### Oxford Nanopore sequencing

Oxford Nanopore sequencing was performed on the GridION sequencer only for the two *Eucalyptus* species (*E. camaldulensis* and *E. rudis*). Prior to library preparation, short fragments were depleted from the extracted HMW DNA using BluePippin with a 30-Kb cut-off or the Short Read Eliminator XL kit (SRE XL, Circulomics, Baltimore, MD, USA) using the manufacturer’s instructions. The size-selected DNA was cleaned-up with AMPure XP beads and then processed into libraries using genomic DNA by ligation protocol (SQK-LSK109, Oxford Nanopore, Oxford, UK). Briefly, DNA (*E. camaldulensis*, 1.3 μg; *E. rudis*, 4 μg) was repaired and end-prepped prior to bead clean-up and adapter ligation. The ligated product is bead cleaned with Long Fragment Buffer (LFB) and eluted. The prepared library (*E. camaldulensis*, 8 fmol; *E. rudis*, 50 fmol) is then loaded onto GridION (FLO-MIN106D).

### Bioinformatics and genome assembly

#### Sequencing data analysis

For PacBio SMRTLink v8.0/v9.0 was used for designing and monitoring sequencing runs and analyzing and managing sequence data. For ONT MinKNOW Core 3.6.0 was used for data acquisition and real-time analysis. Reads were base called using Guppy 3.2.8 from FAST5 files to produce FASTQ files. Statistical analyses were performed using R 3.6.1 [62].

The quality metrics of base called reads were also calculated using LongQC version 1.2 [41]. We applied pb-hifi, pb-sequel, and ont-ligation profiles for PacBio HiFi, PacBio CLR, and Oxford Nanopore datasets, respectively.

#### Genome size estimation

GenomeScope 2.0 [43] was used to estimate, in silico, the genome sizes of both *Eucalyptus* species. The software was run with the following parameters: [*k-mer length = 21, Ploidy = 2, Max k-mer coverage = − 1, average k-mer coverage for polyploid genome = − 1*]. We calculated the *k*-mer distribution, which we then fed to GenomeScope, using JellyFish [63] with the following parameters: [*jellyfish count -C -m 21 -s 1000000000*]. For the peanut genome, we run the *k*-mer analysis using kmc v3.1.2 [64] [*-k21 -m500 -ci1 -cs 100000000*], then kmc_tools [*-cx100000000*] then GenomeScope2 with the following [*ploidy = 4, kmer = 21*].

#### Genome assemblies

We assembled the HiFi data for both *Eucalyptus* species using the newly released assembler hifiasm v0.8 - r279 [45]. We run hifiasm with default settings (*-r3 -a4 -k51 -w51 -f37 -D5.0 -N100 -z0 -m10000000 -p 100000 -n3 -x0.8 -y0.2*). We also separated haplotigs using the purge_dups module in hifiasm using default settings (*-l2 -s0.75 -O1*). Output from hifiasm is two GFA graph files: one for the primary contigs and another for the alternative haplotigs. We converted the GFA files to the FASTA format using gfatools v0.4-r179-dirty [65]. To produce the total assembly statistics, we remerged the primary and alternative haplotigs using seqkit [66].

For PacBio CLR we used the Canu assembler v2 (github r9818) [44]. To force Canu to keep haplotigs separate, we set the following parameters: *batOptions=-dg 3 -db 3 -dr 1 -ca 500 -cp 50*. For speed gains, we run Canu in cluster mode. Where deep coverage was available, we increased the amount of data used in the assembly beyond the default 40x coverage.

#### Polishing of genome assemblies

We polished the CLR-based assemblies using the Arrow algorithm in the gcpp tool from PacBio’s SMRT Link v8.0 stack. First, we aligned the raw CLR data against the initial assembly using pbmm2 v1.1.0, which is a version of Minimap2 [67] adapted to PacBio’s native format. The alignment is then used for consensus calling and polishing using gcpp v1.0.0. We repeated the process for two additional polishing cycles whereby we feed the polished assembly from the previous cycle as the alignment reference in the next cycle. The HiFi-based assemblies do not require additional polishing to the highly accurate starting CCS sequences [44].

#### Genome assemblies’ assessment

We generated comprehensive assembly statistics using QUAST-LG v5.0.2 [68]. To assess the biological integrity of the assemblies, we used BUSCO v3 [69] as a proxy of genome completeness.

#### Haplotig purging

We removed haplotigs for the CLR-based assemblies using purge_dups [70] using default settings. We manually inspected read depth to adjust coverage cut-offs where necessary for best performance. The hifiasm pipeline, on the other hand, is able to separate haplotigs for the HiFi-based assemblies without additional tools (Additional file 1: Fig. S12). We validated the success of this step for all assemblies using BUSCO v3 [69].

## Supplementary Information

**Additional file 1.** Supplementary materials. Includes supplementary results and all supplementary tables (S1-S8) and figures (S1-S12).

**Additional file 2.** Review history.

## Data Availability

The assembled genomes and relevant raw data are available under NCBI BioProject ID PRJNA674723 [71], PRJNA739547 [72], and PRJNA737587 [73] and WGS accessions JAHSVA000000000, JAHSVB000000000, JAHMTN000000000, JAIGYT000000000, and JAIGYU000000000.
