# Supplementary material for: LeafGo: Leaf to Genome, a quick workflow to produce high-quality de novo plant genomes using long-read sequencing technology
Source: Genome Biol. 2021 Sep 3;22:256. doi: 10.1186/s13059-021-02475-z (PMC8414726; doi:10.1186/s13059-021-02475-z)
Supplement: Supplementary file 1 — Additional file 1. Supplementary materials. Includes supplementary results and all supplementary tables (S1-S8) and figures (S1-S12). [file 13059_2021_2475_MOESM1_ESM.pdf]

LeafGo: Leaf to Genome, a quick workflow to produce high-quality *de novo* plant genomes using long-read sequencing technology

Patrick Driguez, Salim Bougouffa, Karen Carty, Alexander Putra, Kamel Jabbari, Muppala Reddy, Richard Soppe, Ming Sin Cheung, Yoshinori Fukasawa, Luca Ermini

Additional file 1

Supplementary Results **3**
Oxford Nanopore Technology Sequencing 3
Genome profiling on unassembled data 4
Phenotypic and in silico identification of the *Eucalyptus* species 5
Estimated assembly ploidy 6
Evaluation of four long-read assemblers on PacBio HiFi data 6
Genome assembly: computational resources 7
LeafGo: cost estimates 8

Supplementary Tables S1-S8 **9**
Table S1: Plants used in this study 9
Table S2: Oxford Nanopore Technology Sequencing Results for the two *Eucalyptus* species 10
Table S3: Impact of two size selection methods on Oxford Nanopore Sequencing 11
Table S4: PacBio sequencing results for eight different plant species 12
Table S5: Correlation between library loading and throughput and N50 14
Table S6: Total (not purged) assembly stats comparison between a selection of HiFi-enabled assemblers 15
Table S7: Comparison of computational resources utilization for the assemblers 16
Table S8: Haplotype-separated assembly stats and BUSCO scores 17

Supplementary Figures S1-S12 **18**
Figure S1: Size selection of ONT libraries 18
Figure S2: Capillary electrophoresis of the HMW DNA from ten plants 19
Figure S3: Pulse field gel electrophoresis of extracted plant HMW DNA 21
Figure S4: Capillary electrophoresis and Long-read Sequencing output of CLR libraries 22
Figure S5 Correlation N50 vs P0% 23
Figure S6: Capillary electrophoresis of prepared HiFi libraries 24
Figure S7: LongQC plots of HiFi data for the two *Eucalyptus* species and *Arachis* 25
Figure S8: LongQC plots of CLR data for two *Eucalyptus* species 26
Figure S9: Genome profiling of the two *Eucalyptus* species and *A. hypogaea* based on HiFi data 27

Figure S10: In silico Taxonomic classification of the two *Eucalyptus* species 28
Figure S11: Timeline from sample extraction to assembly for *A. hypogae*a 29
Figure S12: Summarised workflow for the sequencing data: from platform to purged haploid assembly 30

References **31**

Supplementary Results

Oxford Nanopore Technology Sequencing

We sequenced the two *Eucalyptus* species with four GridION ONT flow cells. Results demonstrated the suitability of our HMW extraction protocol for Oxford Nanopore sequencing (Fig. S1 and Table S2). We called a total of 881,946 passed reads constituting a total of 12.99 Gb for *E. camaldulensis* and 1,144,820 passed reads constituting a total of 27.22 Gb for *E. rudis*. For both samples, the passed reads mean length was > 14.7 Kb, the passed read N50 was > 38.7 Kb and the fraction of bases having QV7 was > 84.1%. The longest reads reached 264,200 and 335,781 bases for *E. camaldulensis* and *E. rudis*, respectively. Our sequencing results are in line with ONT sequencing of plant species, including eucalypts [1,2].

Effect of the size selection method

Oxford Nanopore Technology, unlike PacBio sequencing, has theoretically no upper limit on the maximum read length and thus the N50 parameter takes on particular importance. Previous plant studies showed the impact of size selection on the N50 [1,3], and thus we tested two different size-selection methods: a chemical precipitation approach (Short Read Eliminator, SRE-XL) and a gel-based method (BluePippin with 30 Kb cut) using HMW DNA extracted from the two *Eucalyptus* species. Results showed that both methods are equally able to reduce the amount of smaller DNA fragments (Fig. S1). Despite the precipitation-based approach requiring less laboratory time, the fragment size distribution is more spread, thus retaining more shorter fragments. The sequencing N50s for all libraries were very similar (average 41.4 Kb, range: 37.2 Kb - 43.8 Kb) with no apparent difference between species or size-selection method (Table S3).

Genome profiling on unassembled data

LeafGo used the software package GenomeScope 2.0 [4] to infer the size and the heterozygosity level of the two *Eucalyptus* and the *Arachis* genomes from unassembled HiFi sequenced data. The rationale is that if the genome is heterozygous, then the *k*-mer profile will exhibit a characteristic bimodal distribution, as shown in HiFi genome sequencing data (Fig. S9).

We observed two peaks that were centred at coverage values of 20x and 40x in the case of *E. rudis* and 25x and 50x in the case of *E. camaldulensis* (Fig. S9A, S9B). These values represent both heterozygous *k*-mers that have been sequenced at half the coverage (20x and 25x coverage for *E. rudis* and *E. camaldulensis,* respectively) and homozygous *k*-mers sequenced equally from both alleles (40x and 50x coverage for *E. rudis* and *E. camaldulensis,* respectively). Based on the *k*-mer profiling analyses, the haploid genome size of *E. rudis* was estimated to be 506 Mb while *E. camaldulensis* was estimated at a similar size of 510 Mb with a repeat content for both at around 38-39%. Heterozygosity was relatively high in both species (*E. camaldulensis*: 2.19%; *E. rudis*: 1.57%).

For the allotetraploid peanut genome, two distinct ancestral species underwent a hybridization event and four k-mers topologies are expected (a:a:b:b) [4]. The heterozygosity of *Arachis hypogaea* (expressed as not aaaa) is 10.4% with a k-mer genome size of about 635 Mb (Fig. S9C).

Phenotypic and *in silico* identification of the *Eucalyptus* species

Phenotypic identification of the two *Eucalyptus* species indicated that the two sequenced trees belonged to *Eucalyptus camaldulensis* subspecies *obtusa* and *Eucalyptus rudis* subspecies *rudis*. The species were identified by examination of leaves, flower buds, seed pods, seeds, bark and tree morphology. The phenotypic identification was then confirmed to species level by *in silico* DNA analysis. *In silico* DNA metabarcoding based on 336 complete ITS sequences from different *Eucalyptus* genera indicated that the best hits of our query sequences were *E. camaldulensis* and *E. rudis* for each dataset (Fig. S10).

In order to carry out *in silico* identification we obtained 335 complete ITS sequences representing 304 different *Eucalyptus* genera and 31 unknowns from the PLANiTS dataset version 29-03-2020 [5]. As the dataset did not include the ITS sequence for *E. rudis* we downloaded it separately from NCBI Nucleotide (accession: KT631323.1) bringing the total number of our *Eucalyptus* ITS sequences to 336. We also replaced the ITS sequence for *E. camaldulensis* with the longer version deposited in the NCBI nucleotide database (accession: AF190363.1) which, like the ones for *E. rudis*, also includes partial 18S, full 5.8S and partial 26S rRNA genes.

Once assembled we aligned both *E. camaldulensis* and *E. rudis* genomes against the ITS dataset using blastn v2.7.1 [6] with an E-value cut-off set to 1e-5. We assessed the top hits taking into account percentage identity, length of alignment, mismatches and gaps.

Estimated assembly ploidy

The total assembly size in any genome assembling exercise depends on the ploidy of the genome, level of heterozygosity and how well the assembly tool is able to disentangle the different haplotypes. In an ideal assembly scenario where ploidy is P, we should expect a total assembly size (AS) as AS = Genome Size x P. The purging step, whether part of the assembler or a subsequent analysis, is designed to split the haplotypes as best as possible. In an ideal scenario, primary haploid assembly size should equal that of the alternative set. For homozygous genomes, the assemblers will struggle to distinguish between the homologous contigs and the resulting assembly will be the collapsed haploids with an assembly size equating the genome size. For heterozygous genomes, the purging step is able to recover the alternate haplotypes. Where this is possible, the assembly ploidy is an additional metric that, along with other quality metrics such as BUSCO scores, can determine the completeness of an assembly.

We estimated the assembly ploidy (AP) of the assembly using the following basic formulae: $AP = Smaller\ haploid\ /\ Bigger\ haploid$. The smaller and bigger haploids refer to either the primary or the alternative set of contigs from the haplotigs purging step. This remains an estimate and should be taken with caution as we naively assume that the larger set of haplotigs constitutes a complete haploid n.

Evaluation of four long-read assemblers on PacBio HiFi data

We report a comparison of four long-read sequencing assemblers for performance applied on HiFi PacBio data on three different plant species, *E. rudis*, *E. camaldulensis* and *A. hypogaea*.

For each plant species we *de novo* assembled the genome using hifiasm v0.8 [7], HiCanu v2 [8], Flye v2.8.1 [9] and Wtdbg2 v2.5 [10]. Parameters used by each assembler are listed at the bottom of Tables S6 and S7. The assemblies were mainly compared for contiguity, and computational requirements in terms of time and memory consumption. Results are shown in Tables S6 and S7 revealing that hifiasm outperforms other assemblers.

Genome assembly: computational resources

To help the reader understand the different stages where significant compute resources are needed, we refer to the diagram in Fig. S12. First, we will explain the main resource-intensive steps in the CLR mode of sequencing then compare them with the HiFi ones. Compute resources used by the different HiFi assemblers are also listed in Table S7.

CLR mode

Data Transfer

Data transfer from the sequencer to the compute resource will depend on 1) file sizes (usually 1-2 TB) and 2) the speed of the underlying network, if onsite, and/or the internet speed, if data analysis will be carried out in the cloud. In our case, with an onsite computer cluster a network connectivity of 40 Gb/s & 10Gb/s ports, a typical SMRT cell data transfer takes 3-5 hrs.

Assembly

The biggest bottleneck while assembling CLR data is the error correction and the overlap/trimming step. This step can require significant computational resources especially when coverage is high. With large genomes, this can become intractable. For *E. rudis* (~ 600 Mb), with a sequencing coverage of 50x, the Canu assembler took about 20 hours to finish in cluster mode with a total of 3,444 CPU hours and a maximum RSS memory request peaking at 87 Gb. For *E. camaldulensis* (~ 600 Mb), with a sequencing coverage of 230x, Canu, in cluster mode, took 548 hours (1116 hours with queue wait and debugging) with over 72,491 CPU hours (error correction: 15,280, consensus: 50979, assembling: 6231) and maximum RSS request peaking at 69 Gb.

HiFi mode

Data Transfer

This step is similar to the respective section under CLR as Sequel II generates files of comparable sizes in HiFi and CLR mode.

CCS/HiFi generation

For HiFi, the consensus and error correction are carried out before the assembly step. The improved chemistry and the resulting long polymerase reads have simplified the consensus generation and error correction significantly. This is carried out solely at the level of the ZMW read without needing to overlap reads from other ZMWs. An added advantage is the ability to split/chunk the raw file and distribute the workload on as many compute nodes as physically available without the need for complex mpi coding.

For *E. rudis*, with a single SMRT cell, HiFi generation required 4.5 hours in cluster mode consuming 4,532 CPU hours over 10,305 cores. For *E. camaldulensis*, also with a single SMRT cell, the HiFi step took 5 hours over 11,458 total cores using 7,164 CPU hours. For *A. hypogaea*, with 8 SMRT cells, the HiFi step consumed a cumulative total of 40,425 CPU

hours (average ~5000 CPU hr per SMRT cell) over a total of 138,632 cores (compute cluster). However, CCS calling was done while sequencing was taking place and overall, all HiFi reads from the eight SMRT cells were ready 5 hours after the last cell was transferred (Fig. S11).

Assembly

Assembling the HiFi data with hifiasm needed 1.20 hours (80 minutes) for *E. rudis* (40x coverage) and 2 hours (120 minutes) for the *E. camaldulensis* (51x coverage). We ran hifiasm on a single 40 core node where it consumed 53 and 81 CPU hours for *E. rudis* and *E. camaldulensis*, respectively. In terms of memory, the former peaked at 52 Gb and the latter at 64 Gb.

For *A. hypogaea* (genome size 2.6 Gb; 74x coverage), hifiasm assembled all 8 SMRT cells in 29.8 hours using identical hardware as the two eucalypts. Hifiasm consumed a total of 1,081 CPU hours with maximum memory peaking at 317GB. Refer to Table S7 for a detailed breakdown of HiFi assembly, compute requirements and hardware specifications.

Time & computational resources: *E. rudis & E. camaldulensis vs E. pauciflora* assemblies

A hybrid assembly strategy based on Oxford Nanopore long reads and Illumina short reads sequencing was used to recently assemble the *E. pauciflora* genome [11]. This genome for size and complexity is comparable to the two *Eucalyptus* genomes presented in this study (*E. pauciflora:* 595 Mb; *E. rudis:* 549 Mb; *E. camaldulensis:* 532 Mb).

The two *Eucalyptus* genomes generated in this study were generated consuming about hundred less computational resources than the one produced by the hybrid assembly.
For *E. rudis* and *E. camaldulensis* were used complessively ~2,200 CPU hours (error correction by CCS) and 53/81 CPU hours (hifiasm) respectively. The *E. pauciflora* genome needed ~200,000 CPU hours (error correction by Canu) plus 21,000 CPU hours (MaSuRCA assembler).

The two *Eucalyptus* genomes here produced with the only PacBio technology show more contiguity (*E. rudis:*N50 of 26.0 Mb; *E. camaldulensis:* N50 of 41.4 Mb; *E. pauciflora:* N50 of 3.23 Mb).

LeafGo: cost estimates

For a plant with a one Gb genome, we estimate that within approximately seven days a high quality draft genome assembly can be produced from plant tissue for an estimated consumables and compute cost of US$3,000-US$4,000 [12]. For bigger genome sizes, more libraries and SMRT cells will be required, with an additional estimated consumables cost of at least US$1,300-US$1,800 per SMRT cell.

Supplementary Tables S1-S8

Table S1: Plants used in this study

**Species**	**Common Name**	**Family**	**Plant type**	**Genome Size (Mb)**
Arachis hypogaea L.	Peanut	Fabaceae/ Leguminosae	Annual herbaceous	2700
Brassica rapa	Bok choy/ pak choi	Brassicaceae/ Cruciferae	Biennial herbaceous	370
Distichlis palmeri	Nipa grass	Poaceae	Saltgrass	400*
Eucalyptus camaldulensis	River red gum	Myrtaceae	Hardwood tree	600*
Eucalyptus rudis	Flooded gum	Myrtaceae	Hardwood tree	600*
Pennisetum glaucum	Pearl Millet	Poaceae	Grass	1816
Salicornia bigelovii	Dwarf saltwort	Amaranthaceae	Annual shrub	1300*
Salvadora persica	Toothbrush tree	Salvadoraceae	Small evergreen tree	412
Solanum melongena	Eggplant	Solanaceae	Perennial shrub	1210
Zea mays	Sweetcorn	Poaceae	Grass	2135

***estimation**

Table S2: Oxford Nanopore Technology Sequencing Results for the two *Eucalyptus* species

Statistics on raw data calculated using LongQC. * inferred as average

	Passed reads (QV >=7)		Failed reads (QV <7)		Total reads	
Species	*E. Camaldulensis*	*E. rudis*	*E. Camaldulensis*	*E. rudis*	*E. Camaldulensis*	*E. rudis*
Yield (Gb)	12.99	27.22	0.79	2.82	13.78	30.04
Number of reads	881,946	1,144,820	108,581	193,424	990,527	1,338,244
Q7 bases	86.61%	84.10%	32.67%	37.85%	NA	NA
Longest reads	264,200	335,781	172,390	253,974	264,200	335,781
Mean read length	14,734	23,776	7,276	14,560	*11,005	*19,168
N50	38,717	42,367	27,897	37,413	*33,307	*39,890

Table S3: Impact of two size selection methods on Oxford Nanopore Sequencing

Species	Active pores	Run time (hours)	Total Yield (Gb)	Normalized yield (Mb/ hour)	N50 (Kb)	Size selection method
E. camaldulensis	1229	24	8.14	339.2	37.2	SRE-XL
E. camaldulensis	1095	24	5.64	235.0	43.8	BluePippin 30Kb
E. rudis	1679	48	13.72	285.8	42.8	BluePippin 30Kb
E. rudis	1699	48	16.31	339.8	41.8	SRE-XL

Statistics calculated using MinKNOW Core 3.6.0

Table S4: PacBio sequencing results for eight different plant species

Sample Details	Sequel Platform	Type	Insert Size (Kb)	Movie Time (hours)	Total Bases (Gb)	Q20 Yield (Gb)	Q20 Read Quality (median)	Polymerase av. (bp)	Polymerase N50 (bp)	Subread av. (bp)	Subread N50 (bp)	P0 %	P1 %	P2 %	Control Polymerase Read Length (Mean bp)	Local Base Rate
D. palmeri	I	CLR	30	20	14.1	NA	NA	23094	39464	21004	35435	20	62	18	33896	2.80
D. palmeri	I	CLR	30	20	15.2	NA	NA	21973	37524	20255	34414	13	71	16	46266	2.79
E. camaldulensis	I	CLR	30	20	18.1	NA	NA	27292	47335	23299	38312	19	67	14	39479	2.80
E. camaldulensis	I	CLR	30	20	15.3	NA	NA	22041	37357	20477	34503	12	71	17	44527	2.73
E. rudis	I	CLR	30	20	17.8	NA	NA	27719	47854	24691	40998	21	64	14	37377	2.82
E. rudis	I	CLR	30	20	15.7	NA	NA	23465	41037	22080	38105	15	69	16	40673	2.63
P. glaucum	I	CLR	30	20	11.1	NA	NA	28600	47852	27046	44686	51	42	7	51922	2.57
P. glaucum	I	CLR	30	20	10.3	NA	NA	23480	41756	22570	39976	46	47	7	47919	2.56
P. glaucum	I	CLR	30	10	11.0	NA	NA	24392	41309	23748	39877	44	47	9	36426	2.68
P. glaucum	I	CLR	30	20	13.8	NA	NA	24430	40484	23388	38522	34	58	7	47332	2.41
P. glaucum	I	CLR	30	10	10.2	NA	NA	19362	34312	19063	33654	34	54	11	30748	2.43
P. glaucum	I	CLR	30	20	9.7	NA	NA	19121	29486	18512	28583	38	53	10	50240	2.62
P. glaucum	I	CLR	30	10	8.1	NA	NA	17372	29929	16978	28915	43	48	9	37545	2.61
P. glaucum	I	CLR	30	10	3.9	NA	NA	14747	21877	14524	21522	57	28	15	31185	2.64
P. glaucum	I	CLR	30	20	8.9	NA	NA	29054	45656	27336	42461	61	33	7	48680	2.49
P. glaucum	I	CLR	30	20	3.3	NA	NA	24691	44270	22557	40287	66	16	18	42502	2.44
P. glaucum	I	CLR	30	20	3.6	NA	NA	23435	42508	21491	38828	76	18	6	33458	2.74
*P. glaucum**	I	CLR	30	20	14.8	NA	NA	22964	38087	20031	33199	21	65	14	40927	2.77
S. bigelovii	I	CLR	30	20	17.0	NA	NA	31492	49431	26832	41558	32	56	12	45873	2.83
S. persica	I	CLR	30	20	4.3	NA	NA	28105	46406	26166	42980	75	18	6	34346	2.89
S. persica	I	CLR	30	20	14.2	NA	NA	20041	35828	18734	33322	12	73	15	45315	2.61
Z. mays	I	CLR	30	20	12.5	NA	NA	22897	38381	21333	35909	27	58	15	39190	2.63
E. camaldulensis	II	CLR	30	15	195.4	NA	NA	28350	45997	25489	39777	11	86	3	31888	2.01
E. rudis	II	CLR	30	30	150.6	NA	NA	27593	42816	22857	35313	30	68	2	53880	2.05
E. rudis	II	CLR	30	30	140.8	NA	NA	24789	36623	21494	32383	28	71	2	52398	2.07
S. persica	II	CLR	30	15	183.7	NA	NA	27773	44723	24753	38085	14	83	3	35769	2.11
D. palmeri	II	HiFi	18	30	113.9	7.6	33	107849	199058	17750	22156	86	13	0	61220	2.21
D. palmeri	II	HiFi	18	30	356.3	23.0	33	96533	189596	15300	19603	52	46	2	63774	2.25
E. camaldulensis	II	HiFi	17	30	477.3	28.7	33	88261	175407	14568	17673	29	68	3	56714	2.20
E. rudis	II	HiFi	17	30	317.2	22.1	32	101976	195495	17921	21714	60	39	1	55468	2.22
S. persica	II	HiFi	16	30	455.4	29.4	36	118199	200898	15723	17626	51	48	1	59746	2.25
A. hypogaea L.	II	HiFi	17	30	374.1	27.6	28	73951	149609	18431	19981	35	63	2	43151	1.7758
A. hypogaea L.	II	HiFi	16	30	295.3	20.8	31	78403	164781	19595	20710	52	47	1	37158	1.89
A. hypogaea L.	II	HiFi	16	30	324.5	24.6	30	82874	161622	18585	19716	50	49	1	41790	1.83
A. hypogaea L.	II	HiFi	17	30	311.2	23.1	30	86081	165657	19837	20876	54	45	1	38382	1.87
A. hypogaea L.	II	HiFi	17	30	299.8	22.6	30	77252	153809	18871	20216	50	48	1	38192	1.78
A. hypogaea L.	II	HiFi	17	30	291.3	22.4	30	80672	161924	18717	20234	54	45	1	37893	1.82

A. hypogaea L.	II	HiFi	16	30	356.8	25.1	30	81258	166416	19042	20154	43	55	2	38801	1.89
A. hypogaea L.	II	HiFi	16	30	354.0	25.3	30	83141	168325	18789	19929	45	53	1	42480	1.89

* Representative sample of sheared gDNA for library preparation

Table S5: Correlation between library loading and throughput and N50

Cells shaded in red represent significant results (p-value ≤ 0.05).

Spearman's rho	P0%	P1%	P2%	Longest Subread N50 (bp)	Total Bases (Gb)	Polymerase N50 (bp)
**P0%**	1	-0.98	-0.63	0.42	-0.84	0.26
**P1%**	-0.98	1	0.52	-0.39	0.86	-0.23
**P2%**	-0.63	0.52	1	-0.42	0.39	-0.3
**Longest Subread N50 (bp)**	0.42	-0.39	-0.42	1	0	0.93
**Total Bases (Gb)**	-0.84	0.86	0.39	0	1	0.22
**Polymerase N50 (bp)**	0.26	-0.23	-0.3	0.93	0.22	1
**p-value**	**P0%**	**P1%**	**P2%**	**Longest Subread N50 (bp)**	**Total Bases (Gb)**	**Polymerase N50 (bp)**
**P0%**	1	0.00005	0.0018	0.0499	0.00005	0.2463
**P1%**	0.00005	1	0.0129	0.0717	0.00005	0.306
**P2%**	0.0018	0.0129	1	0.0548	0.0763	0.1752
**Longest Subread N50 (bp)**	0.0499	0.0717	0.0548	1	0.9861	0.00005
**Total Bases (Gb)**	0.00005	0.00005	0.0763	0.9861	1	0.3159
**Polymerase N50 (bp)**	0.2463	0.306	0.1752	0.00005	0.3159	1

Table S6: Total (not purged) assembly stats comparison between a selection of HiFi-enabled assemblers

Versions used: HiCanu v2, Flye v2.8.1, Wtdbg2 v2.5, hifiasm v0.8

Species	Tool	Size (Mb)	no. contigs	Largest	N50/L50	N90/L90
E. rudis	hifiasm [∈]	975.1	1005	61.8	10.7/14	1.1/129
	HiCanu [∅]	1009.3	2112	35.4	6.5/43	0.6/215
	Flye [Σ]	732.4	13217	9.7	~0.2/751	0.02/5135
	Wtdbg2 [Δ]	494.7	3577	3.6	~0.5/245	0.06/1355
E. camaldulensis	hifiasm [∈]	1053.1	1131	69.1	18.9/14	1.6/114
	HiCanu [∅]	1064.6	1751	32.9	10.5/28	2.1/111
	Flye [Σ]	881.7	16002	6.5	~0.2/1008	0.02/6621
	Wtdbg2 [Δ]	532.1	4290	4.0	~0.5/253	0.05/1619
A. hypogaea	hifiasm [∈]	2674.5	2802	90.3	40.1/23	10.3/69
	HiCanu [∀]	3819.0	41958	59.2	11.3/85	~0.3/25152
	Flye [Σ]	2540.9	1519	24.9	6.9/113	1.6/394
	Wtdbg2 [∇]	2181.4	26416	1.6	~0.2/3097	~0.04/13210

[∈] hifiasm options: `-l 2`

[∅] Additional HiCanu options: `genomeSize=680m batOptions="-eg 0.01 -sb 0.01 -dg 6 -db 6 -dr 1 -ca 50 -cp 5"`

[∀] Additional HiCanu options:: `genomeSize=2.7g`

[Δ] Wtdbg2 options: `-x ccs -g 600m`

[∇] Wtdbg2 options: `-x ccs -g 2.7g`

[Σ] Flye options: `--keep-haplotypes`

Table S7: Comparison of computational resources utilization for the assemblers

Species	Tool	Mode	Hardware Specs	Time (hr)	Requested Cores	CPU Hrs	Max RSS (GB)
E. rudis	hifiasm ∈	1 node	Intel Xeon Gold 6148: 40 cores<br>RAM: 350GB	1.3	40	53	52
	HiCanu ⌀	cluster	Heterogeneous	5.9	1644	503	21
	Flye Σ	1 node	Intel Xeon Gold 6148: 40 cores<br>RAM: 350GB	5.3	40	214	72
	Wtdbg2 ∆	1 node	Intel Xeon Gold 6148: 40 cores<br>RAM: 350GB	0.9	40	34	32
E. camaldulensis	hifiasm ∈	1 node	Intel Xeon Gold 6148: 40 cores<br>RAM: 350GB	2.0	40	81	63
	HiCanu ⌀	cluster	Heterogeneous	8.0	2138	856	18
	Flye Σ	1 node	Intel Xeon Gold 6148: 40 cores<br>RAM: 350GB	5.6	40	223	60
	Wtdbg2 ∆	1 node	Intel Xeon Gold 6148: 40 cores<br>RAM: 350GB	1.0	40	39	41
A. hypogaea	hifiasm ∈	1 node	Intel Xeon Gold 6148: 40 cores<br>RAM: 350GB	29.8	40	1081	317
	HiCanu ∀	cluster	Heterogeneous	32.6	14585	11743	51[2]
	Flye Σ[1]	1 node	Intel Xeon Gold 6246: 48 cores<br>RAM: 3TB	84.7	40	3391	575
	Wtdbg2 ∇1	1 node	Intel Xeon Gold 6246: 48 cores<br>RAM: 3TB	66.7	40	2669	321

∈ hifiasm options: `-l 2`
⌀ Additional HiCanu options: `genomeSize=680m batOptions="-eg 0.01 -sb 0.01 -dg 6 -db 6 -dr 1 -ca 50 -cp 5"`
∀ Additional HiCanu options:: `genomeSize=2.7g`
∆ Wtdbg2 options: `-x ccs -g 600m`
∇ Wtdbg2 options: `-x ccs -g 2.7g`
Σ Flye options: `--keep-haplotypes`

[1] Flye & wtdbg2 for *A. hypogaea* failed to run on nodes with 350GB and 470GB of RAM
[2] Hicanu RAM: HiCanu in Cluster mode splits the jobs which explains the low MaxRSS

Table S8: Haplotype-separated assembly stats and BUSCO scores

P: primary haplotigs/contigs which refer to the longer of the contigs that belong to a region of high heterozygosity. **A**: alternative haplotigs/contigs are the other non-primary haplotigs/contigs. **S**: single complete BUSCO, **D**: duplicate complete BUSCO.

**Species**	**Assembly type**	**Primary/ Alternative**	**Total Length (Mb)**	**N50 (Mb)**	**Longest (Mb)**	**Assembly Ploidy (2N)ß**	**BUSCO**		
							**Complete %**	**Fragment %**	**Missing %**
E. rudis	HiFi ∂	P	549.4	36.0	61.8	1.77N	97.3 [S:91.7,D:5.6]	1.1	1.6
		A	425.7	3.5	10.7		87.2 [S:83.4,D:3.8]	1.3	11.5
	CLR Δ	P	518.4	16.3	33.7	1.77N	96.3 [S:92.4,D:3.9]	1.7	2.0
		A	399.9	~0.4	3.88		73.4 [S:66.7,D:6.7]	2.3	24.3
E. camaldulensis	HiFi ø	P	532.9	41.4	69.1	1.98N	97.2 [S:93.9,D:3.3]	0.9	1.9
		A	520.2	4.1	19.3		94.2 [S:89.7,D:4.5]	1.0	4.8
	CLR Δ	P	523.5	29.3	58.1	1.92N	97.3 [S:93.2,D:4.1]	0.9	1.8
		A	570.2	2.3	12.3		93.5 [S:75.8,D:17.7]	1.2	5.3
A. hypogaea	HiFi	P c	2623.2	42.3	90.3	NA	97.5 [S:14.6,D:82.9]	0.4	2.1
		A	51.2	0.03	2.6		NA	NA	NA

ß Estimated assembly ploidy (AP). Refer to the haplotig purging section in Methods.

∂ Haplotigs were purged using the purge module within hifiasm [7].

Δ gcpp-polished assembly was purged using purge_dups [13] .

ø HiFi assembly was purged using purge_dups as the hifiasm purging module did not produce the best results.

c Duplicate BUSCOs in the purged *A. hypogaea* assembly are high. This is expected and is in support of a high quality assembly and purging step. The source of the duplication is the two subgenomes.

Supplementary Figures S1-S12

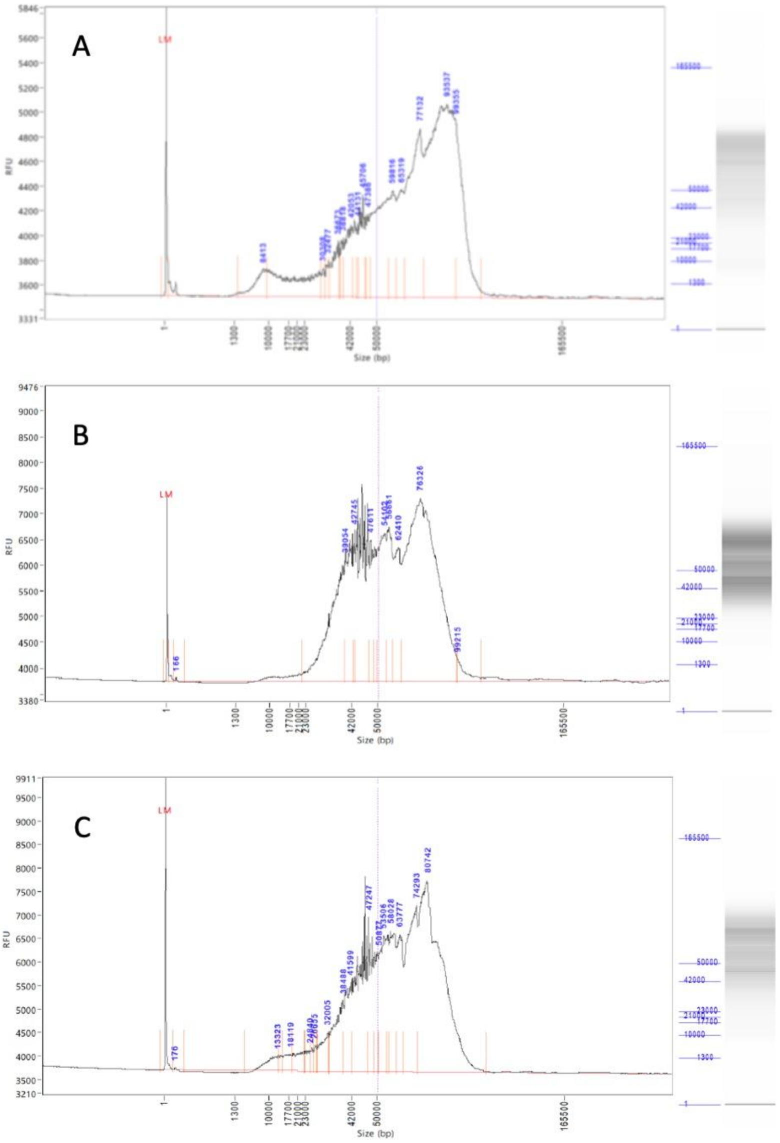

Figure S1: Size selection of ONT libraries

Capillary electrophoresis (FEMTO Pulse, Method: FP-1002E22 – Extended gDNA 165kb) of gDNA (A), BluePippin 30 Kb selected DNA (B), and SRE-XL size selected DNA (C), from *Eucalyptus rudis*.

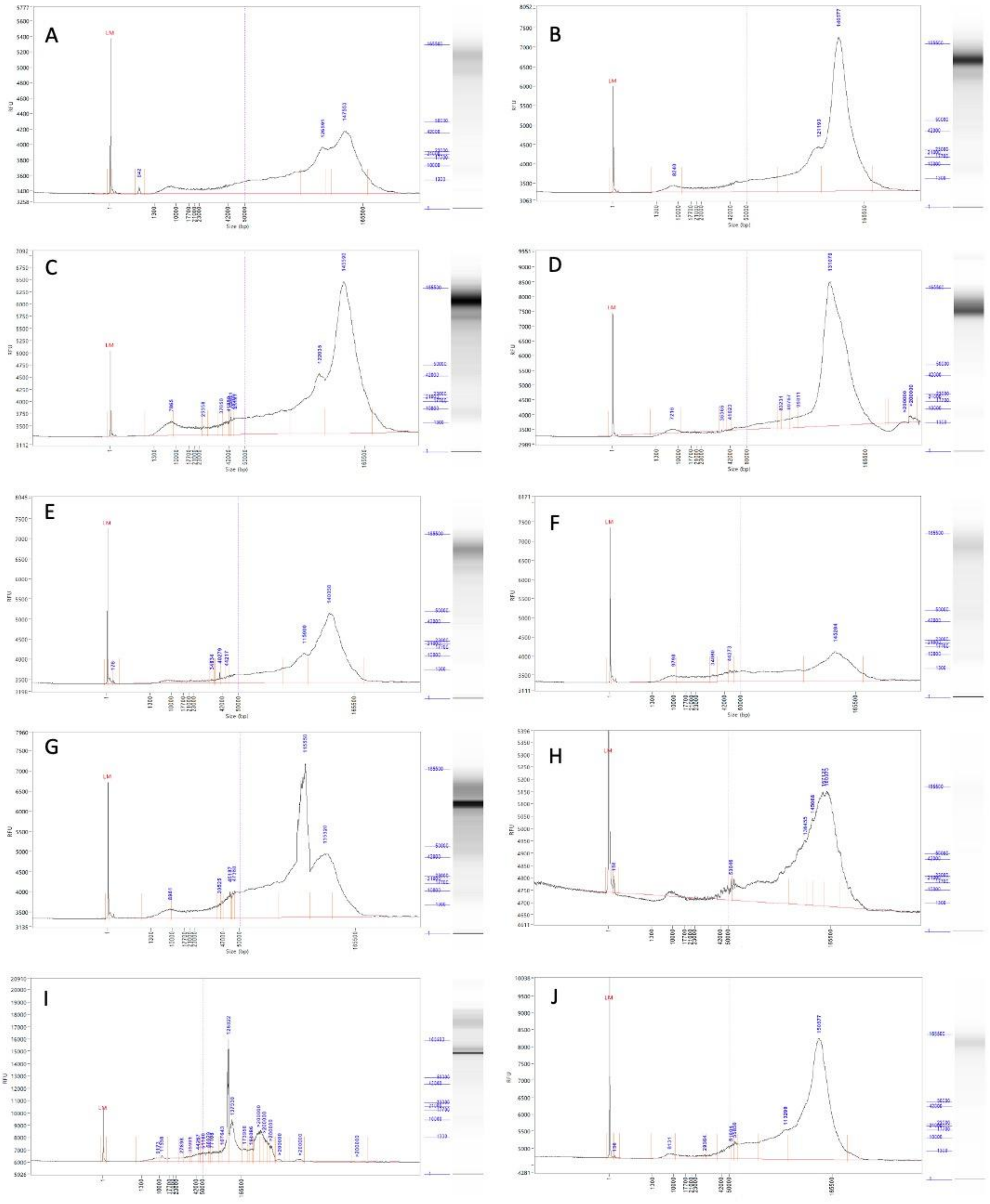

Figure S2: Capillary electrophoresis of the HMW DNA from ten plants

FEMTO 165 Kb ladder analysis (Method: FP-1002E22 – Extended gDNA 165 Kb) of extracted DNA from *Eucalyptus camaldulensis* (A); *Eucalyptus rudis* (B); *Distichlis palmeri* (C); *Salvadora persica* (D); *Pennisetum glaucum* (E); *Zea mays* (F); *Salicornia bigelovii* (G); *Arachis hypogaea* L. (H), *Brassica rapa* subsp. *chinesis* (I) and *Solanum melongena* (J).

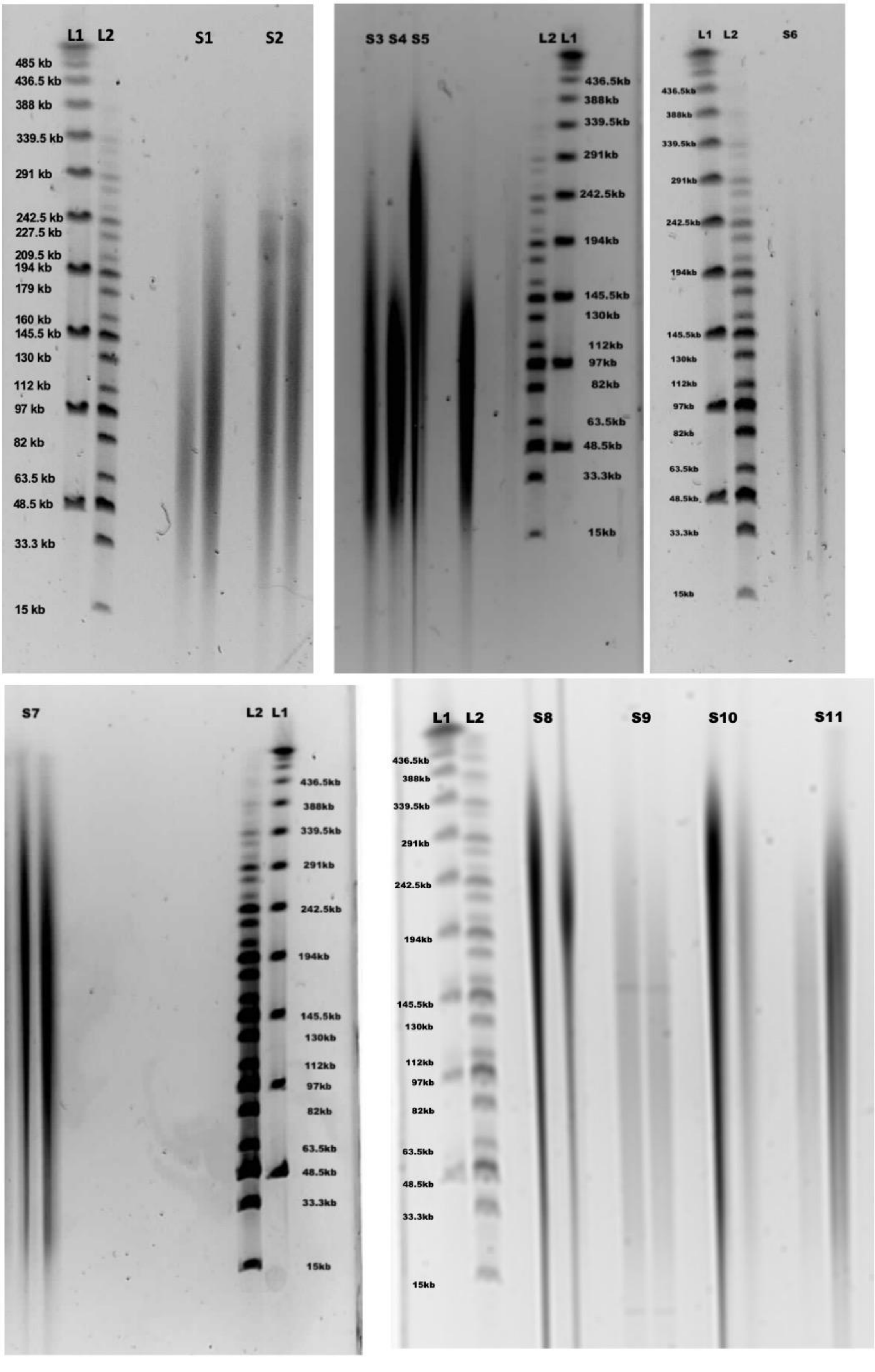
L1 L2 S1 S2
485 kb
436.5 kb
388 kb
339.5 kb
291 kb
242.5 kb
227.5 kb
209.5 kb
194 kb
179 kb
160 kb
145.5 kb
130 kb
112 kb
97 kb
82 kb
63.5 kb
48.5 kb
33.3 kb
15 kb
S3 S4 S5 L2 L1
436.5kb
388kb
339.5kb
291kb
242.5kb
194kb
145.5kb
130kb
112kb
97kb
82kb
63.5kb
48.5kb
33.3kb
15kb
L1 L2 S6
436.5kb
388kb
339.5kb
291kb
242.5kb
194kb
145.5kb
130kb
112kb
97kb
82kb
63.5kb
48.5kb
33.3kb
15kb
S7 L2 L1
436.5kb
388kb
339.5kb
291kb
242.5kb
194kb
145.5kb
130kb
112kb
97kb
82kb
63.5kb
48.5kb
33.3kb
15kb
L1 L2 S8 S9 S10 S11
436.5kb
388kb
339.5kb
291kb
242.5kb
194kb
145.5kb
130kb
112kb
97kb
82kb
63.5kb
48.5kb
33.3kb
15kb

Figure S3: Pulse field gel electrophoresis of extracted plant HMW DNA

PFGE Image of HMW DNA, Lambda PFG Ladder (NEB N0341S) (L1), Midrange PFG Marker (NEB N0342S) (L2), Extracted DNA from *Eucalyptus rudis* (S1), *Eucalyptus camaldulensis* (S2), *Zea Mays* (S3), *Distichlis palmeri* (S4), *Salvadora persica* (S5), *Salicornia bigelovii (S6), Pennisetum glaucum* (S7), *Arachis hypogaea* L. (S8 and S10), *Brassica rapa* subsp. *chinesis* (S9) and *Solanum melongena* (S11).

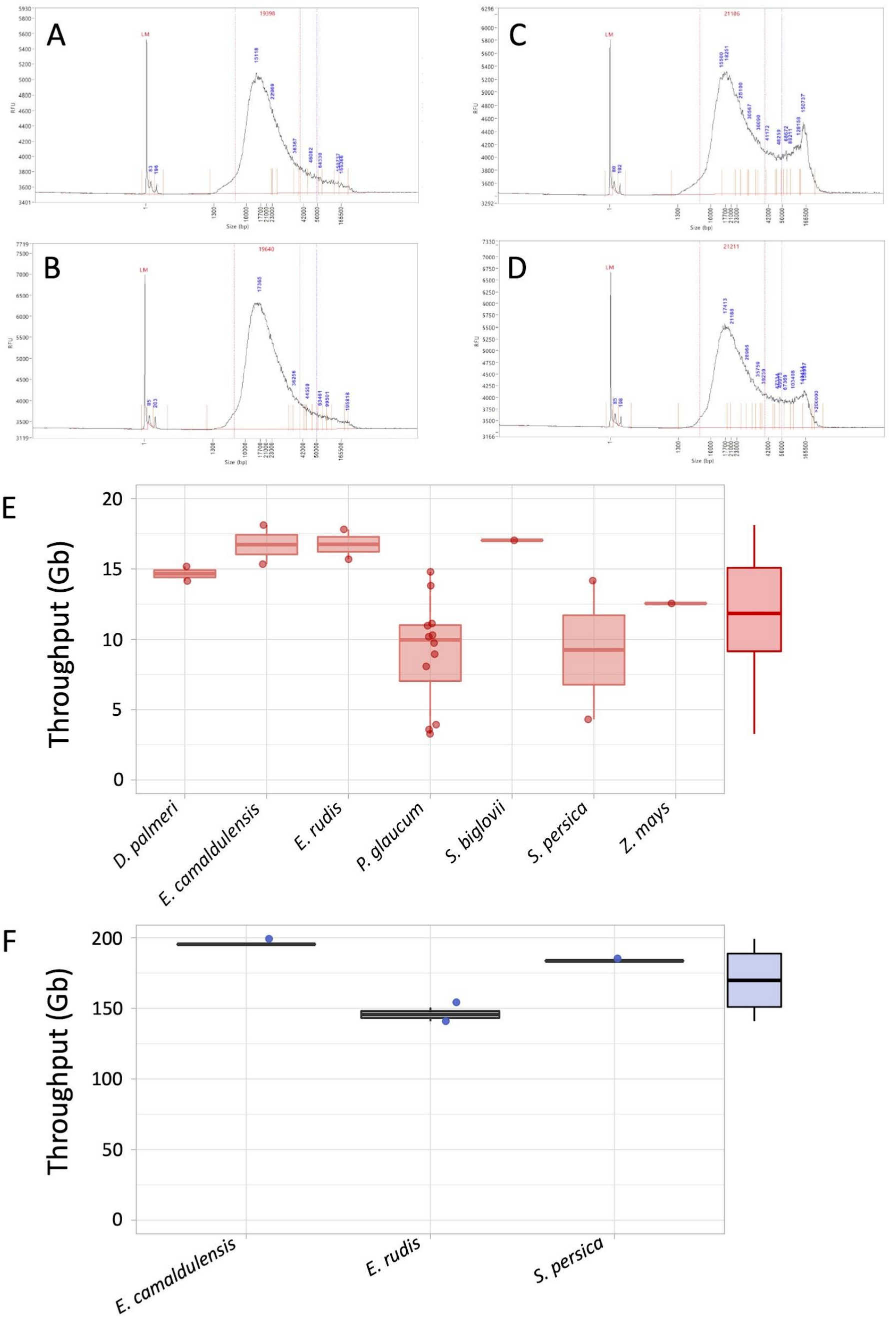

Figure S4: Capillary electrophoresis and Long-read Sequencing output of CLR libraries

A-D: Capillary Electrophoresis; FEMTO Pulse (Method: FP-1002E22 – Extended gDNA 165 Kb), results for PacBio CLR library of *Distichlis palmeri* (A), *Salvadora persica* (B), *Eucalyptus rudis* (C), *Eucalyptus camaldulensis* (D). E, F: The total throughput per species of CLR libraries on Sequel I (E) and Sequel II (F). The average for all data is plotted along the margin.

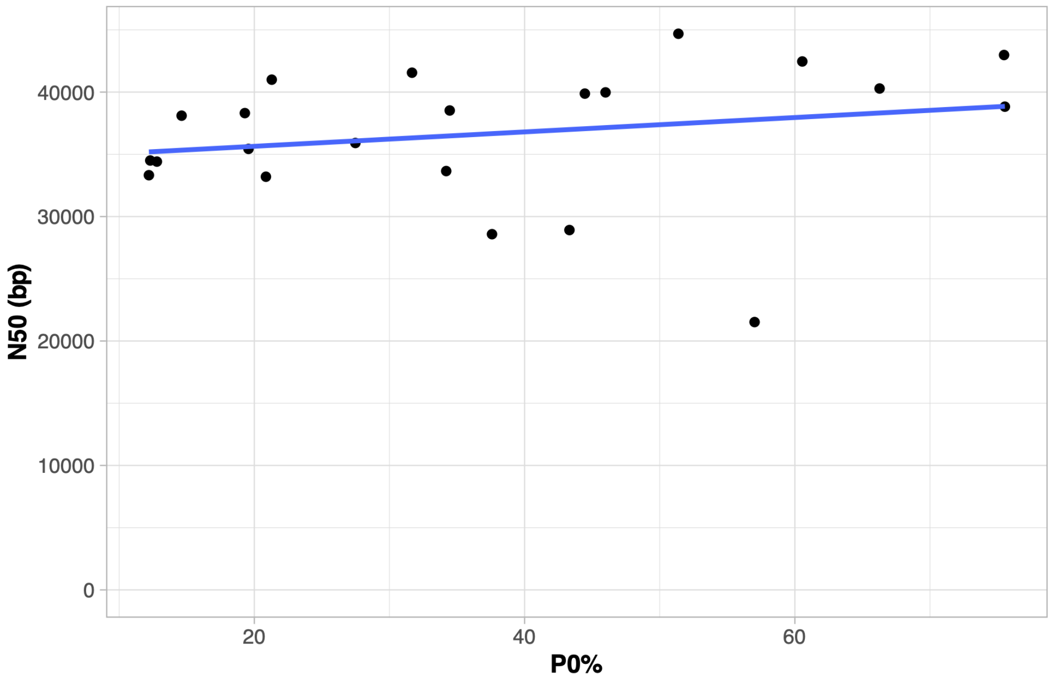

Figure S5 Correlation N50 vs P0%

Correlation between ZMW occupancy or library underloading (P0%) and subread N50 of CLR libraries sequenced with Sequel I. Spearman's rho = 0.42, p = 0.0499.

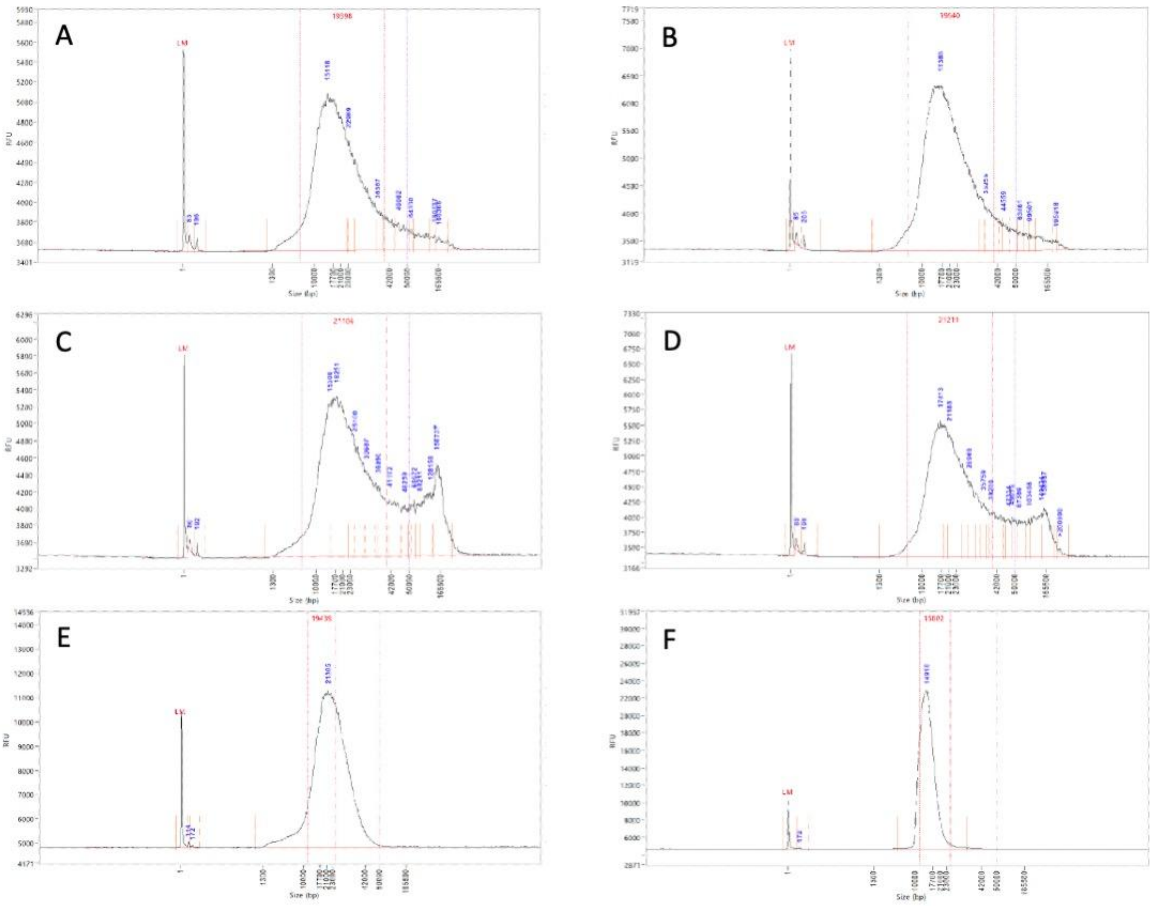

Figure S6: Capillary electrophoresis of prepared HiFi libraries

FEMTO Pulse (Method: FP-1002-22 – gDNA 165 Kb), results for PacBio HiFi library of *Eucalyptus camaldulensis* before (A) and after size selection (B), *Eucalyptus rudis* before (C) and after size selection (D), and *Arachis hypogaea* L. before (E) and after size selection (F). The *Eucalyptus* samples were sheared with g-TUBES and size-selected with AMPure PB beads; *A. hypogaea* L. was sheared with Megaruptor 2 and size-selected with BluePippin.

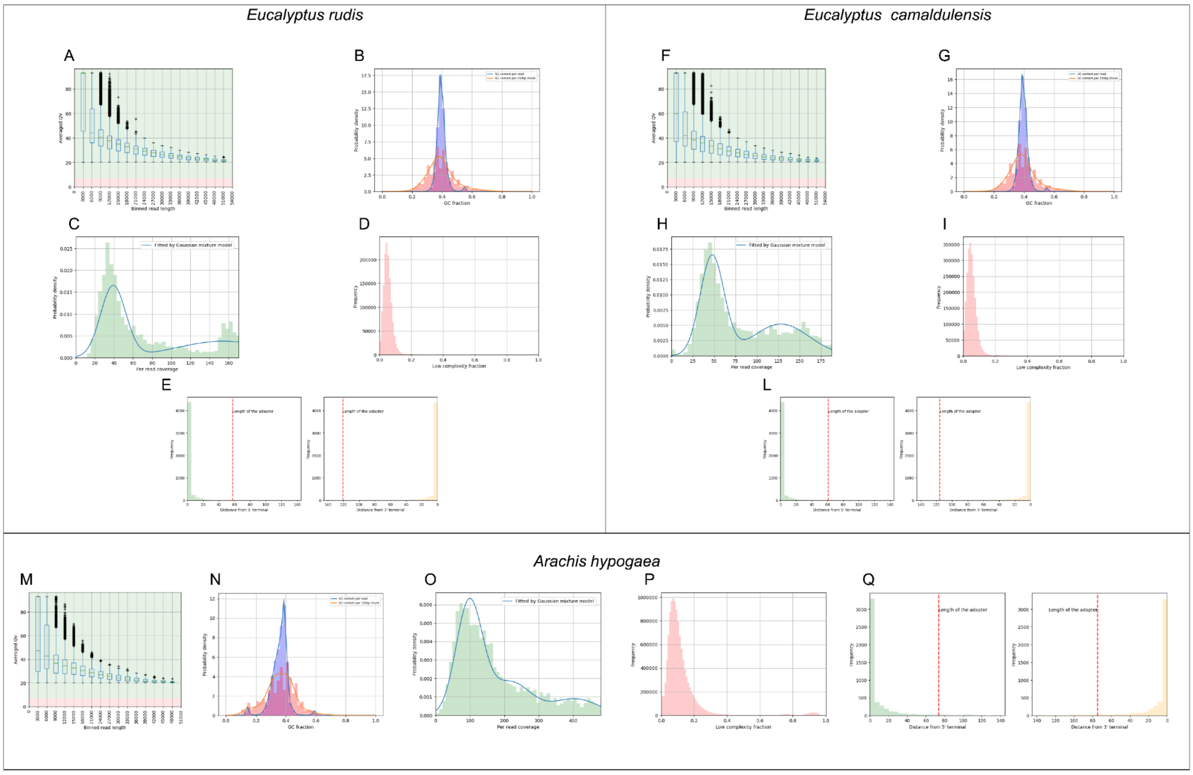

Figure S7: LongQC plots of HiFi data for the two *Eucalyptus* species and *Arachis*

Sample data QC plots for PacBio HiFi dataset of *E. rudis*, *E. camaldulensis* and *A. hypogaea* generated by LongQC. (A, F, M): read-QV distribution alongside length, (B, G, N): GC content, (C, H, O) estimated depth, (D, I, P): sequence complexity and (E, L, Q): flanking region analysis.

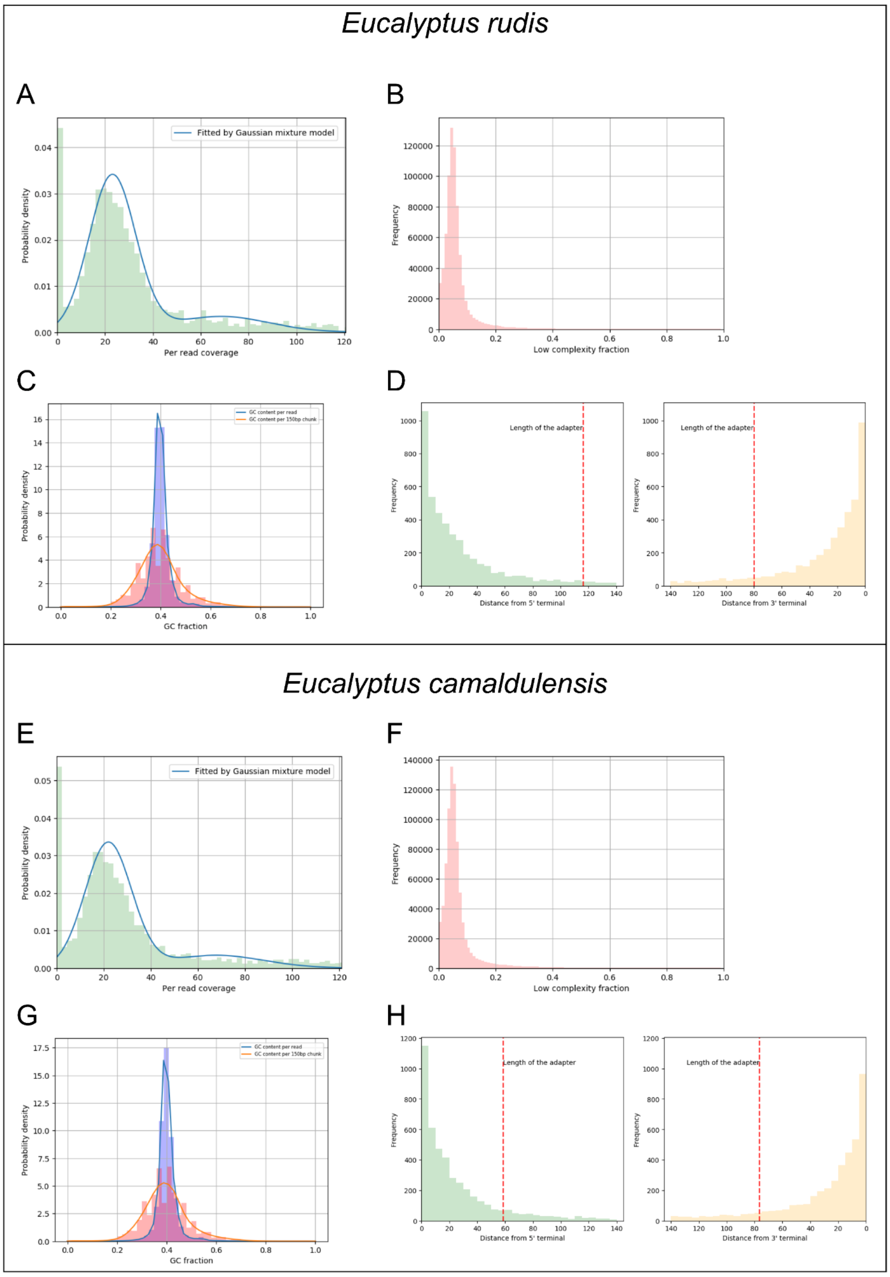

Figure S8: LongQC plots of CLR data for two *Eucalyptus* species

Sample data QC plots for PacBio CLR dataset of *E. rudis* and *E. camaldulensis* generated by LongQC. (A,E): estimated depth, (B,F): sequence complexity, (C,G): GC content and (D,H): flanking region analysis.

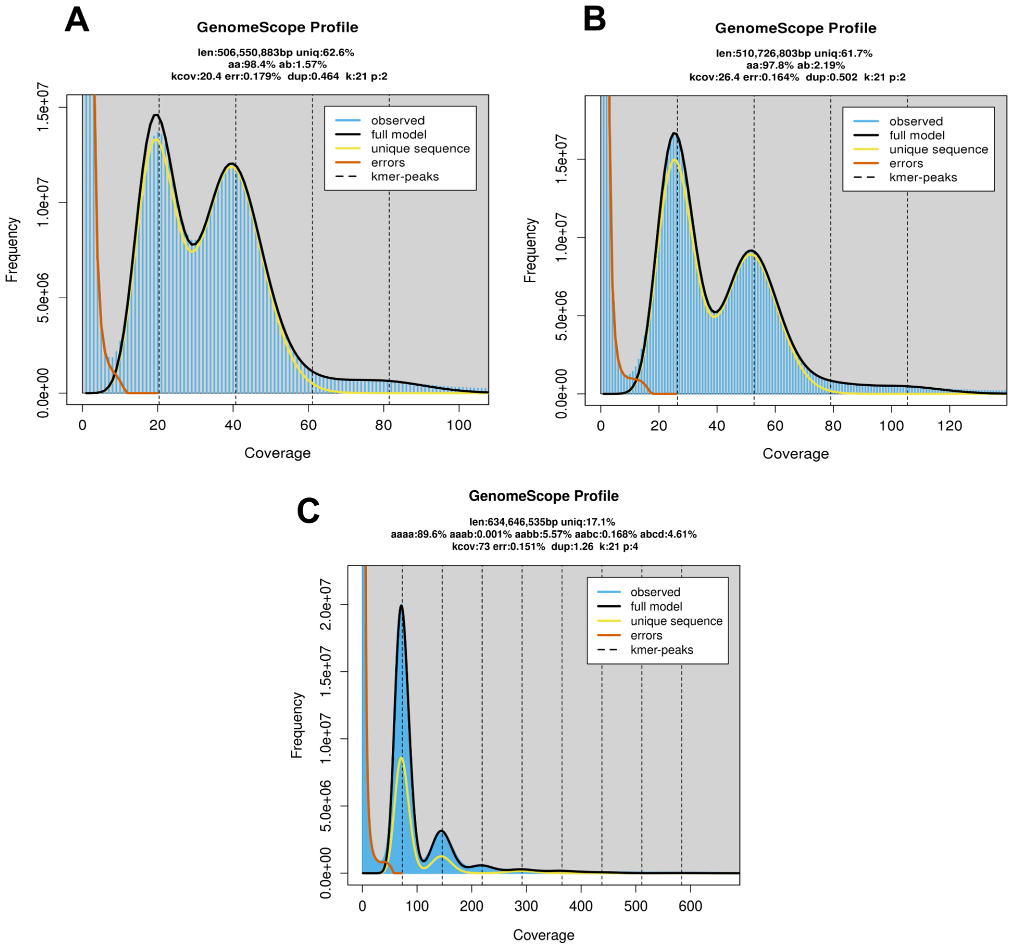

Figure S9: Genome profiling of the two *Eucalyptus* species and *A. hypogaea* based on HiFi data

k-mer profiles, fitted models, and estimated parameters for the diploid genomes of (A) *E. rudis* (heterozygosity = 1.5%, repeat = 37.4%),and (B) *E. camaldulensis* (heterozygosity = 2.1%, repeat = 38.3%), and (C) *A. hypogaea* (heterozygosity = 12.5%, repeat = 82.9%). It should be noted that Genomescope2 reports the genome size of a single chromosome set. So the estimated genome size is *len* multiplied by 4 giving 2.54Gb.

Legend: *len* is the estimated genome size. *Uniq* is the size of the non-repeat content. *aa,ab*: homo and heterozygosity level for a diploid genome. *aaaa, aabb, aabc, abcd* refer to heterozygosity for an allotetraploid genome. *Kcov* is estimated coverage for heterozygous *k*-mers. *Err*: read error rate. *Dup*: PCR duplication. *P*: genome ploidy.

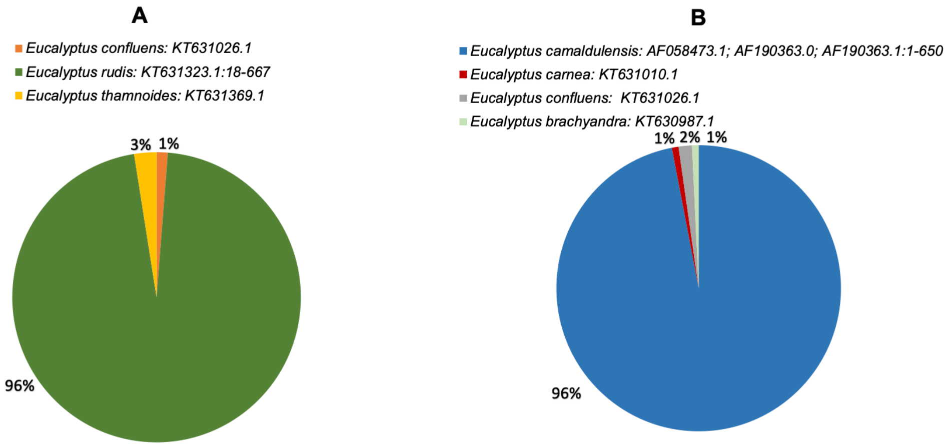

Figure S10: *In silico* Taxonomic classification of the two *Eucalyptus* species

Identification of *E. rudis* (A) and *E. camaldulensis* (B) by DNA metabarcoding analysis of both *Eucalyptus* genomes against the ITS dataset. Each pie chart shows the proportion of species identified by the top hit of each query.

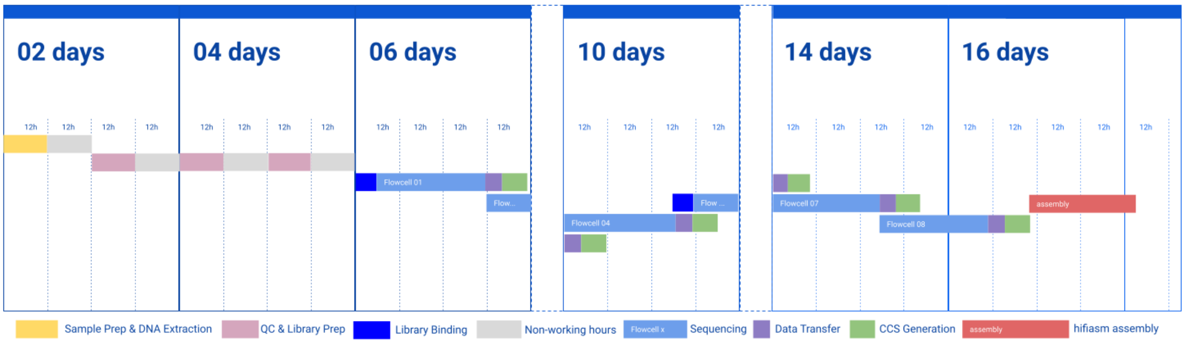

Figure S11: Timeline from sample extraction to assembly for *A. hypogaea*

Sample preparation should be the same for other plants regardless of genome size. Sequencing time depends on the number of SMRT cells needed (genome size dependent) and the available sequencing platforms. The above timeline is based on a single Sequel II scenario. Assembly time will also depend on the throughput (*i.e.* number of SMRT cells).

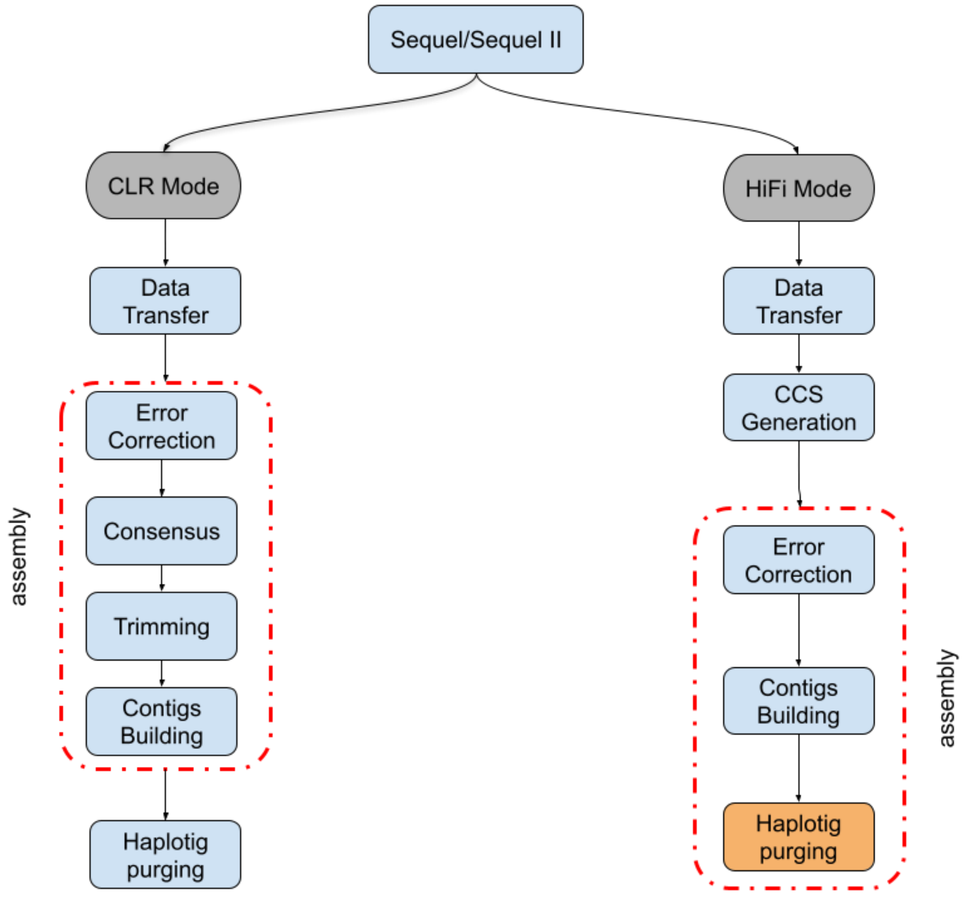

Figure S12: Summarised workflow for the sequencing data: from platform to purged haploid assembly

Major differences between CLR and HiFi modes are: 1) the “CCS Generation” step in the HiFi mode (right) which is essentially error correction and consensus calling steps on produced sequencing data (often performed during assembly). 2) The assembly stage in the HiFi mode (right) is more simplified with a very fast error correction step as the total HiFi bp size is smaller than CLR subread size. The haplotig purging step is often a separate step from assembly except for hifiasm.

References

1. Schalamun M, Nagar R, Kainer D, Beavan E, Eccles D, Rathjen JP, et al. Harnessing the MinION: An example of how to establish long-read sequencing in a laboratory using challenging plant tissue from *Eucalyptus pauciflora*. Mol Ecol Resour. 2019;19:77–89.
2. Choi JY, Lye ZN, Groen SC, Dai X, Rughani P, Zaaijer S, et al. Nanopore sequencing-based genome assembly and evolutionary genomics of circum-basmati rice. Genome Biol. 2020;21:21.
3. Belser C, Istace B, Denis E, Dubarry M, Baurens F-C, Falentin C, et al. Chromosome-scale assemblies of plant genomes using nanopore long reads and optical maps. Nat Plants. Nature Publishing Group; 2018;4:879–87.
4. Ranallo-Benavidez TR, Jaron KS, Schatz MC. GenomeScope 2.0 and Smudgeplot for reference-free profiling of polyploid genomes. Nat Commun. Nature Publishing Group; 2020;11:1432.
5. Banchi E, Ametrano CG, Greco S, Stanković D, Muggia L, Pallavicini A. PLANiTS: a curated sequence reference dataset for plant ITS DNA metabarcoding. Database [Internet]. 2020 [cited 2020 Dec 22];2020. Available from: https://doi.org/10.1093/database/baz155
6. Altschul SF, Gish W, Miller W, Myers EW, Lipman DJ. Basic local alignment search tool. J Mol Biol. England; 1990;215:403–10.
7. Cheng H, Concepcion GT, Feng X, Zhang H, Li H. Haplotype-resolved de novo assembly using phased assembly graphs with hifiasm. Nat Methods. Nature Publishing Group; 2021;18:170–5.
8. Nurk S, Walenz BP, Rhie A, Vollger MR, Logsdon GA, Grothe R, et al. HiCanu: accurate assembly of segmental duplications, satellites, and allelic variants from high-fidelity long reads. Genome Res. 2020;30:1291–305.
9. Kolmogorov M, Yuan J, Lin Y, Pevzner PA. Assembly of long, error-prone reads using repeat graphs. Nat Biotechnol. Nature Publishing Group; 2019;37:540–6.
10. Ruan J, Li H. Fast and accurate long-read assembly with wtdbg2. Nat Methods. Nature Publishing Group; 2020;17:155–8.
11. Wang W, Das A, Kainer D, Schalamun M, Morales-Suarez A, Schwessinger B, et al. The draft nuclear genome assembly of *Eucalyptus pauciflora*: a pipeline for comparing de novo assemblies. GigaScience. 2020;9:1–12.
12. Logsdon GA, Vollger MR, Eichler EE. Long-read human genome sequencing and its applications. Nat Rev Genet. 2020;21:597–614.
13. Guan D, McCarthy SA, Wood J, Howe K, Wang Y, Durbin R. Identifying and removing haplotypic duplication in primary genome assemblies. Bioinformatics. Oxford Academic; 2020;36:2896–8.
