# Supplementary material for: LeafGo: Leaf to Genome, a quick workflow to produce high-quality de novo plant genomes using long-read sequencing technology
Source: Genome Biol. 2021 Sep 3;22:256. doi: 10.1186/s13059-021-02475-z (PMC8414726; doi:10.1186/s13059-021-02475-z)
Supplement: Supplementary file 2 — Additional file 2. Review history. [file 13059_2021_2475_MOESM2_ESM.docx]

Review History

**First round of review**

**Reviewer 1**

**Were you able to assess all statistics in the manuscript, including the appropriateness of statistical tests used?**

No, I do not feel adequately qualified to assess the statistics.

**Comments to author:**

Long-reads sequencing technologies are under fast development in recent years. For genome assembly, longer reads would span across bigger regions, including highly repetitive or heterozygous regions, and thus allowing to produce more complete and accurate assemblies. By applying long-read sequencing from different platforms on seven plant species, along with high quality high molecular weight (HMW) DNA extraction method, the authors' group verify the feasibility of these methods on plant genomic sequencing and assembly. Based on this, a workflow called Leaf to Genome (LeafGo) was introduced, which can de novo assemble plant genome from tissues within as short as seven days. With this workflow, they successfully assembled two high-quality eucalypt genomes. This work would give good guidance for plant genome assembly in the new sequencing era.

Major comments:

1. The author has mentioned that one of the biggest stumbling blocks for adopting long-read sequencing technology is that high quality of HMW DNA is hard to extract, especially from plants that contain lots of phenols and polysaccharose. However, in this article, we can't find how the authors solve this problem. If the author has done some innovative modification to the Qiagen Genomic protocol, especially steps that help improve the quality and yield of HMW DNA should be highlighted in the article. In addition, I think the species tested in this study is not representative enough for all plant kingdom which belongs to only four families, and perhaps evidence of high quality DNA isolation from Solanaceae, Leguminosae, or Cruciferae species could be included to further support the method.

2. The major job in this research is LeafGo workflow and its application on E. rudis and E. camaldulensis genome assembly. For this reason, it would be better to move the works done on other species (including DNA extraction and sequencing) after these two, and also try to simplify this part. In addition, the biological problem to solve and the conclusion of this part were not clear and should be stated. A concise diagram would be enough for data showing, so it would better to simplify the text to make the main point clear.

3. For the assembly methods, a comparison to other assembly methods (eg. Wtdbg2, Flye et al.) could be included to help justify the proposed method.

4. The advantages of LeafGo workflow are simple, fast and low consuming. There should be a more direct comparison with previously published assemblies done with long-read sequencing platforms to illustrate this point, including aspects like time, money, labor, and computing resources consuming.

5. P9 285-286 "compatibility with long read ONT sequencing was demonstrated". We can't see the demonstration in the article. The workflow is somehow not complete for ONT sequencing because of lacking the corresponding assembly result.

Minor comments:

1. It will be better to verify the assemblies using other sequencing data, for example: using NGS data to evaluate the base-level accuracy and using HiC data to inspect the mis-assembly.

2. P3 line 64: "differing" should be "different".

3. P3 line75: comma should be added between "repetitive" and "making".

4. P4 line 99: delete the word "centers".

5. P6 line 173: "12,99 Gb" should be "12.99 Gb" ?

6. Table S4: the first row-name should be "Species" instead of "Specie".

7. Figure S7 and Figure S8 are relative and can adopt same arrangement. The figure legend of GC content (G,B in Figure S7, CG in Figure S8) should move apart to avoid covering the curses.

8. Unified writing style or units should be adopted. Eg. P4 line 128: kb, but in line129: Kb.

9. Some citation types should be modified into "Journal" rather than "Internet". Eg. Reference 19, 22,33, etc.

**Reviewer 2**

**Were you able to assess all statistics in the manuscript, including the appropriateness of statistical tests used?**

Yes, and I have assessed the statistics in my report.

**Comments to author:**

LeafGo claims to be "a quick workflow to produce high-quality de novo genomes using long-read sequencing technology." The manuscript presents two draft genome assemblies produced using this workflow: Eucalyptus rudis and Eucalyptus caamaldulensis.

1)	In line 84 of the manuscript, the authors indicate that seven plant species were used to develop the LeafGo workflow. Aside from raw sequencing data, there is little other mention of five of the seven species. Was there any assembly performed on these five additional species? If so, where are those assembly statistics and how long did those genomes take from start to finish?

2)	The claim that de novo plant genomes can be produced within seven days is caveated with a statement of "haploid genome of 0.6 to 1.0 Gb" in line 261. Is there any information on how long a larger or more complex genome would take? Three of the seven species mentioned have genomes larger than 1.0 Gb so this data should theoretically be available.

3)	Each plant is different in its composition. Has this protocol been used on plant species beyond the seven mentioned in this paper?

4)	There is no novel pipeline or program presented in this paper. Every program or pipeline used (with perhaps the exception of DNA extraction) has been previously described.

Ultimately, this pipeline seems to be promising for smaller plant genomes. The promise is unclear for larger or more complex (for example: polyploid) genomes or plants whose DNA is harder to extract. I feel that the claim that de novo plant genomes can be produced in seven days is presented without sufficient evidence. The Eucalyptus genomes presented look good, and some analysis of them (and perhaps other Eucalyptus genomes already available) could warrant a nice genome analysis paper of them.

**Reviewer 3**

**Were you able to assess all statistics in the manuscript, including the appropriateness of statistical tests used?**

There are no statistics in the manuscript.

**Comments to author:**

General comments

The authors present a workflow, LeafGo, to assemble plant genomes with long-read sequencing. It is a nice demonstration that a single DNA extraction protocol works on multiple plant species, and a confirmation that recent long-read sequencing and assembly methods work well. It is clearly written, easy to follow, and I have no concern about the soundness of the study. However the novelty and potential impact on the field is limited by the absence of thorough benchmarks of either wet lab or dry lab components. The manuscript is more akin to a technical report than a highly-novel research study.

Specific comments

1. It was not clear to me initially what LeafGo was (a DNA extraction protocol? an assembly pipeline?) because if an assembly project is divided in the steps 1) DNA extraction, 2) library prep and sequencing, 3) bioinformatics assembly, steps 2 and 3 in LeafGo may contain lots of different things (Pacbio, ONT, CLR, HiFi, Canu, Miniasm,...). Since "LeafGo" may be lots of things, I am not sure it is useful to have a name for it, as referring to "LeafGo" will still be ambiguous.

2. The strength of the manuscript lies in the DNA extraction protocol, a modified version of the Qiagen genomic DNA protocol, which seems applicable to leaf tissue from many plant species. There are a number of protocols floating around (in publications, protocols.io, Nanopore Community website) and one that works on many species is very nice. This section could be further developed in the results, explaining how it departs from the manufacturer's recommendations, and where specifically it succeeds where other methods fail, or if they don't fail how it avoids using toxic reagents.

3. For the library prep and sequencing as well as the bioinformatics assembly, it seems that standard protocols work fine, which is good to see but confirmatory rather than novel results. As only the two eucalyptus species (of the 7 plant samples) are carried through to assembly, it is not known whether the assembly protocol is universal like the DNA extraction. One can envision that larger genomes, higher repetitiveness and higher heterozygosity may cause problems, which is where the forefront of genome assembly is. Did the authors attempt assembly of the other genomes? The benchmark between Pacbio CLR and HiFi reads is contrived, as it is already known the newer and higher quality HiFi reads are better building blocks for assemblies. They also benefit from newer dedicated software (hifiasm). In this aspect the manuscript is not helping researchers select their sequencing technology (Pacbio HiFi vs Nanopore, as in recent publication 10.1093/gigascience/giaa146) or analysis software (hifiasm is not benchmarked against competitors). These benchmarks are difficult to do in a field that evolves so fast (both sequencing tech and algorithms), and require constant updates. However since the authors generated Nanopore data, it could be used for some limited benchmarking.

**Authors Response**

**Point-by-point responses to the reviewers’ comments:**

Reviewer #1:

Long-reads sequencing technologies are under fast development in recent years. For genome assembly, longer reads would span across bigger regions, including highly repetitive or heterozygous regions, and thus allowing to produce more complete and accurate assemblies. By applying long-read sequencing from different platforms on seven plant species, along with high quality high molecular weight (HMW) DNA extraction method, the authors' group verify the feasibility of these methods on plant genomic sequencing and assembly. Based on this, a workflow called Leaf to Genome (LeafGo) was introduced, which can de novo assemble plant genome from tissues within as short as seven days. With this workflow, they successfully assembled two high-quality eucalypt genomes. This work would give good guidance for plant genome assembly in the new sequencing era.

Major comments:

1. The author has mentioned that one of the biggest stumbling blocks for adopting long-read sequencing technology is that high quality of HMW DNA is hard to extract, especially from plants that contain lots of phenols and polysaccharose. However, in this article, we can't find how the authors solve this problem. If the author has done some innovative modification to the Qiagen Genomic protocol (1.1), especially steps that help improve the quality and yield of HMW DNA should be highlighted in the article. In addition, I think the species tested in this study is (1.2) not representative enough for all plant kingdom which belongs to only four families, and perhaps evidence of high quality DNA isolation from Solanaceae, Leguminosae, or Cruciferae species could be included to further support the method.

*(1.1)*

*We thank the reviewer for this comment. We did not remake the protocol, we just modified some steps, as described in the material and methods section. A step-by-step protocol link is also provided (page 12, line 424). However, one of the features of the protocol is the cleaning steps which purify the DNA. Hence in the Methods page 12, lines 412-413 we rewrote the sentence as follow: “Repeated washing in ethanol, a major modification of our protocol, has been shown to improve DNA purity without a decrease in yield”.*

*(1.2)*

*The reviewer commented that “the species tested in this study is not representative enough for all plant kingdoms which belong to only four families” suggesting to isolate DNA from Solanaceae, Leguminosae or Cruciferae species.*

*We really appreciate this suggestion as it gives consistency and robustness to the manuscript. We extracted High Molecular Weight DNA from three different plants belonging to each one of the family suggested: eggplant (Solanum melongena; family: Solanaceae; genome size: 1,200 Mb), pak choi (Brassica rapa; family: Brassicaceae or cruciferae; genome size: 370 Mb) and peanut (Arachis hypogaea; family: Fabaceae or Leguminosae;genome size: ~2,600 Mb). The high-quality DNA isolation is shown in supplementary figure S1 and S2. Due to financial reasons, it was not possible to sequence both eggplant and pak choi but we sequenced and de novo assembled the complex tetraploid genome of peanut (A. hypogaea). The high quality of the assembly is demonstrated in the assembly stats of table (Table 1) and in Figure 3*

2. (2.1) The major job in this research is LeafGo workflow and its application on E. rudis and E. camaldulensis genome assembly. For this reason, it would be better to move the works done on other species (including DNA extraction and sequencing) after these two, and also try to simplify this part. (2.2) In addition, the biological problem to solve and the conclusion of this part were not clear and should be stated. (2.3) A concise diagram would be enough for data showing, so it would better to simplify the text to make the main point clear.

*(2.1)*

*We thank the reviewer for raising this point, and we agree with him/her. In order to broaden the application of LeafGo, we extracted DNA from three different families (please refer to the previous point) and we de novo assembled the complex tetraploid genome of the peanut (A. hypogaea). This allowed us to shift the focus from the Eucalyptus species and widen the scope of the paper to include additional results and discussion for peanut.*

*(2.2)*

*We understand that the reviewer could not formulate a clear idea about the biological/methodological problem that we set out to solve and we appreciate this input.*

*The crucial biological/methodological problem of LeafGo is to achieve the best operating conditions for assembling high quality de novo genomes using only one technology. Our goal was achieved by considering the overall procedure, and modifying and optimizing each single step for consistent results.*

*We have now clarified our goal and the biological/methodological problem in the introduction (page 3, lines 68-73) and we also discuss it (page 10, lines 312-317).*

*We think that this manuscript is more suitable as a method article rather than a research article and therefore we propose it as a method article.*

*(2.3)*

*We modified the text (we added a new genome) and revised the structure to help the reader to better follow the manuscript. In particular we added subheadings in the genome assembly section of the results which is particularly difficult to follow. In addition, the map of the computational resources needed is challenging to follow and we added a diagram (Figure S11). We have also included a further flowchart of the timeline for our workflow from extraction to assembly (Figure S12).*

3. For the assembly methods, a comparison to other assembly methods (eg. Wtdbg2, Flye et al.) could be included to help justify the proposed method.

*We thank the reviewer for this comment and we agree. In fact, as our work evolved, we have compared different assemblers and even the same assemblers with different configurations. Initially no HiFi-tuned assemblers were available. In PAG2019, HiCanu was announced by its author and so we used the early release. Much later, and as we began writing our work, hifiasm also became available and was tested and adopted by us in the workflow. We initially contemplated including a comparison of assemblers in this work despite the fact that this may have been beyond the scope of our article and any information we included could quickly become outdated, especially considering the rate that current assemblers are evolving.*

*However, to make our work more comprehensive and justify the choice of assemblers we have cited, an exhaustive comparison of assemblers for long-read sequencing (Murigneux et al “Comparison of long-read methods for sequencing and assembly of a plant genome”, GigaScience, 9, 2020, 1–11). This GigaScience study compared four different assemblers for PacBio CLR including the one suggested by the reviewer (Flye) and also one used by us (Canu) plus two more assemblers (Redbean, and Raven).*

*Furthermore, we agree with the reviewer that adding a tool comparison between HiFi assemblers could be of value to the community since little can be found in recently published literature, especially from the non-developer community. Taking on board the reviewer comment, we compared assembly performance between the following HiFi assemblers: hifiiasm,wtdbg2, Flye, and HiCanu. We include our comparison in supplementary material (“Evaluation of four long-read assemblers on PacBio HiFi data”) and we addede two tables (Tables S7 and S8). Our comparison shows that hifiasm outperforms the other assemblers.*

4. The advantages of LeafGo workflow are simple, fast and low consuming. There should be a more direct comparison with previously published assemblies done with long-read sequencing platforms to illustrate this point, including aspects like time, money, labor, and computing resources consuming.

*We acknowledge the point the reviewer makes but it is difficult to make this kind of comparison. LeafGo is a complete workflow from sample preparation and HMW DNA extraction to the bioinformatics assembly and to our knowledge, at the present, there is no such workflow in the scientific literature. However, we added a possible comparison with the recent assembly of Eucalyptus pauciflora, another similar size and complexity genome, which was generated throughout a hybrid assembly strategy, (ONT long reads + Illumina short reads). This therefore comparison supports the strength of LeafGo (page 8, lines 258-264; Added file within the secrion: “Genome assembly: computational resources”).*

5. P9 285-286 "compatibility with long read ONT sequencing was demonstrated". We can't see the demonstration in the article. The workflow is somehow not complete for ONT sequencing because of lacking the corresponding assembly result.

*We are sorry for the oversight, and we agree with the reviewer, this statement is incomplete. Initially, we set out to compare CLR to the newly released HiFi protocol. Then, we reckoned that the extraction protocol could prove useful for ONT as well.*

*Although our results are similar to other ONT sequencing of plant genomes, the throughput we obtained was not sufficient (table S7) for a proper genome assembly. Considering the limitation of funding and previous comparisons between ONT and PacBio data (i.e.Lang et al, Comparison of the two up-to-date sequencing technologies for genome assembly: HiFi reads of Pacific Biosciences Sequel II system and ultralong reads of Oxford Nanopore, GigaScience, Volume 9, Issue 12, December 2020, giaa123, https://doi.org/10.1093/gigascience/giaa123) we decided to not proceed further.*

*We amended our oversights and we modified as follows:*

*In the results section page 6, line 164 :*

*“Results demonstrated the suitability of our HMW extraction protocol for Oxford Nanopore sequencing”*

*In the discussion section (the above statement, page 10, lines 318-319):*

*“The use of one sequencing technology such as PacBio is a distinctive advantage of LeafGo, although compatibility of the extracted HMW DNA with long-read ONT is also demonstrated”*

Minor comments:

1. It will be better to verify the assemblies using other sequencing data, for example: using NGS data to evaluate the base-level accuracy and using HiC data to inspect the mis-assembly.

*We thank the reviewer for this comment as we have, in fact, considered this ourselves and we agree that this may improve the assembly. However our primary aim is to pave the way for the use of a single technology. Indeed, it would have been ideal to inspect potential misassembles using a technology such as HiC but due to funding limitations, we are unable to sequence the three provided genomes with NGS or HiC.*

2. P3 line 64: "differing" should be "different".

*Corrected*

3. P3 line75: comma should be added between "repetitive" and "making".

*Corrected*

4. P4 line 99: delete the word "centers".

*We changed the whole sentence*

5. P6 line 173: "12,99 Gb" should be "12.99 Gb" ?

*Corrected*

6. Table S4: the first row-name should be "Species" instead of "Specie".

*Corrected*

7. Figure S7 and Figure S8 are relative and can adopt same arrangement. The figure legend of GC content (G,B in Figure S7, CG in Figure S8) should move apart to avoid covering the curses.

*Corrected*

8. Unified writing style or units should be adopted. Eg. P4 line 128: kb, but in line129: Kb.

*Corrected*

9. Some citation types should be modified into "Journal" rather than "Internet". Eg. Reference 19, 22,33, etc.

*Corrected*

Reviewer #2:

LeafGo claims to be "a quick workflow to produce high-quality de novo genomes using long-read sequencing technology." The manuscript presents two draft genome assemblies produced using this workflow: Eucalyptus rudis and Eucalyptus camaldulensis.

1) In line 84 of the manuscript, the authors indicate that seven plant species were used to develop the LeafGo workflow. Aside from raw sequencing data, there is little other mention of five of the seven species. Was there any assembly performed on these five additional species? If so, where are those assembly statistics and how long did those genomes take from start to finish?

*We thank the reviewer for pointing this out. For project and data retention reasons we could not assemble all the genomes from the species we extracted DNA and sequenced. The five additional species include two species with an estimated genome size (Distichlis palmeri: about 400 Mb and Salvadora persica about 412 Mb) similar to the Eucalyptus species we sequenced, and on this basis assembling time and resources should be similar or less to those used for the Eucalyptus species. In addition, we plan publishing papers for the Distichlis and Salvadora genomes .*

*The other three species (Salicornia bigelovii, Pennisetum glaucum and Zea mays) have a more complex genome with an estimated size of 1300 Mb, 1800 Mb and 2100 Mb, respectively. We address the reviewer’s point by the de novo assembly of the complex tetraploid genome of peanut (A. hypogaea) with a size of about 2623 Mb. See Figure 3. We also provided all the assembling statistics and resources needed (see Table 1, supplementary Tables S5, S7 and S8).*

2) The claim that de novo plant genomes can be produced within seven days is caveated with a statement of "haploid genome of 0.6 to 1.0 Gb" in line 261. Is there any information on how long a larger or more complex genome would take? Three of the seven species mentioned have genomes larger than 1.0 Gb so this data should theoretically be available.

*We de novo assembled the tetraploid genome of the peanut (A. hypogaea). More complex genomes require more days due mainly to the necessity of producing more sequencing data and thus more SMRT cells to run with PacBio Sequel II. The possibility of running more SMRT cells in parallel using different platforms will reduce the sequencing time for larger genomes; for a company or a big institute, this option may be possible.*

*We agree with the reviewer that time information for larger genomes is valuable. We provide the time needed from DNA extraction to the assembled genome for A. hypogaea. (Figure S12) and we describe in details time and computational resourced needed in the supplementary material (section: Genome assembly: computational resources). In addition, in the results section (page 10, lines 285-295) we describe the time estimations.*

3) Each plant is different in its composition. Has this protocol been used on plant species beyond the seven mentioned in this paper?

*We agree that we could have tested the protocol with many more plant species but this task would have required much more resources not least in terms of manpower, consumables, funding (restricted) etc. However, this protocol has been used for the seven plant species in the first draft of the manuscript and now includes three additional species from three different families in the revised version (see above also). Additionally, direct feedback from three other laboratories has shown our protocol was successfully used to extract HMW DNA from at least three species (not shown here). Having said that, we have tried to address this comment and a similar suggestion from reviewer 1 (comment 1.2) by adding three plants (from three different families: Solanaceae, eggplant, Solanum melongena; Leguminosae, peanut, Arachis hypogaea; Cruciferae, pak choi, Brassica rapa) to further support the method. We successfully extracted HMW DNA for all (see Supplementary figures: S1 and S2).*

4) There is no novel pipeline or program presented in this paper. Every program or pipeline used (with perhaps the exception of DNA extraction) has been previously described.

*We acknowledge this comment and we also agree with the reviewer. However, the aim of the study is not to provide new software or a new technology. We aim to provide a complete workflow and a guideline to assemble high quality de novo genomes in a short time. On this ground the complete pipeline is novel and the combining of different resources is novel. Compared to multi-technique assemblies ours is unique.*

Ultimately, this pipeline seems to be promising for smaller plant genomes. The promise is unclear for larger or more complex (for example: polyploid) genomes or plants whose DNA is harder to extract. I feel that the claim that de novo plant genomes can be produced in seven days is presented without sufficient evidence. The Eucalyptus genomes presented look good, and some analysis of them (and perhaps other Eucalyptus genomes already available) could warrant a nice genome analysis paper of them.

*We thank the reviewer for raising this ultimate and important point and we really appreciate this suggestion. We provided a de novo assembly of the peanut (Arachis hypogaea) allotetraploid genome (size: ~2.6 Gb) which gives consistency and additional robustness to the manuscript. Please refer also to points 1 and 2 and reviewer 1, point .*

Reviewer #3:

The authors present a workflow, LeafGo, to assemble plant genomes with long-read sequencing. It is a nice demonstration that a single DNA extraction protocol works on multiple plant species, and a confirmation that recent long-read sequencing and assembly methods work well. It is clearly written, easy to follow, and I have no concern about the soundness of the study. However the novelty and potential impact on the field is limited by the absence of thorough benchmarks of either wet lab or dry lab components. The manuscript is more akin to a technical report than a highly-novel research study.

Specific comments

1. It was not clear to me initially what LeafGo was (a DNA extraction protocol? an assembly pipeline?) because if an assembly project is divided in the steps 1) DNA extraction, 2) library prep and sequencing, 3) bioinformatics assembly, steps 2 and 3 in LeafGo may contain lots of different things (Pacbio, ONT, CLR, HiFi, Canu, Miniasm,...). Since "LeafGo" may be lots of things, I am not sure it is useful to have a name for it, as referring to "LeafGo" will still be ambiguous.

*We are sorry for the lack of clarity and we agree with the reviewer that LeafGo is a lot of things and missing a proper explanation could be misleading and generate confusion. We now properly define LeafGo in the introduction (page 4, lines 90-93):*

*“This paper provides the rationale for the adoption of LeafGo, a workflow that combines different resources to generate de novo genomes of different plants. LeafGo consists of a robust HMW DNA extraction method, library preparation and sequencing approach, and genome assembly suggestions.”*

*In addition, we found out that the repetitive usage of the LeafGo term over the manuscript could be overwhelming and we decided to carefully reduce the use of it.*

2. The strength of the manuscript lies in the DNA extraction protocol, a modified version of the Qiagen genomic DNA protocol, which seems applicable to leaf tissue from many plant species. There are a number of protocols floating around (in publications, protocols.io, Nanopore Community website) and one that works on many species is very nice. This section could be further developed in the results, explaining how it departs from the manufacturer's recommendations, and where specifically it succeeds where other methods fail, or if they don't fail how it avoids using toxic reagents.

*We thank the reviewer for this comment. Please refer to the reviewer 1, comment 1.1.*

3. For the library prep and sequencing as well as the bioinformatics assembly, it seems that standard protocols work fine, which is good to see but confirmatory rather than novel results. As only the two eucalyptus species (of the 7 plant samples) are carried through to assembly, it is not known whether the assembly protocol is universal like the DNA extraction. One can envision that larger genomes, higher repetitiveness and higher heterozygosity may cause problems, which is where the forefront of genome assembly is. Did the authors attempt assembly of the other genomes? The benchmark between Pacbio CLR and HiFi reads is contrived, as it is already known the newer and higher quality HiFi reads are better building blocks for assemblies. They also benefit from newer dedicated software (hifiasm). In this aspect the manuscript is not helping researchers select their sequencing technology (Pacbio HiFi vs Nanopore, as in recent publication 10.1093/gigascience/giaa146) or analysissoftware (hifiasm is not benchmarked against competitors). These benchmarks are difficult to do in a field that evolves so fast (both sequencing tech and algorithms), and require constant updates. However since the authors generated Nanopore data, it could be used for some limited benchmarking.

*We completely agree with the reviewer that this is a fast evolving field, we are using here the most advanced developments. On this basis our paper uses the latest advances in sequencing technology and it is one of the first works using hifiasm, since the package was published. Many researchers in the plant community are not fully aware of the benefits of HiFi sequencing for genome assembly and here, we demonstrate this on three different genomes (two different Eucalypt diploid genomes and one complex tetraploid Peanut genome). At the present only few articles have been released emphasizing the benefits of HiFi sequencing, many of those cited in this manuscript. Beyond HiFi, we included CLR sequencing and we used the insert N50, as a sequencing quality control measure for assessing extracted HMW DNA; to our knowledge, this is a novelty QC for assessing HMW DNA extractions. In addition, while HiFi sequencing doesn’t require large insert sizes (< 20 Kb) , starting with HMW DNA with a large mode of fragment length is essential. Furthermore, future iterations of HiFi sequencing may need larger insert libraries.*

*We thank the reviewer for the comment and we followed his/her suggestion, both in terms of benchmarking and assembling other genomes.*

*We assembled the complex tetraploid genome of peanut (Arachis hypogaea, genome size: 2,6 Gb). The high quality of the assembling is shown in Figure 3.*

*To make our work more comprehensive and justify the choice of assemblers we have cited, an exhaustive comparison of assemblers for long-read sequencing (Murigneux et al “Comparison of long-read methods for sequencing and assembly of a plant genome”, GigaScience, 9, 2020, 1–11).*

*However, we compared assembly performance between the following HiFi assemblers: hifiiasm,wtdbg2, Flye, and HiCanu. We include our comparison in supplementary material (“Evaluation of four long-read assemblers on PacBio HiFi data”) and we addede two tables (Tables S7 and S8). Our comparison shows that hifiasm outperforms the other assemblers.*

*Please see reviewer 1, point 3.*

**Second round of review**

**Reviewer 1**

The authors have successfully addressed all of my concerns.

**Reviewer 2**

I would like to thank the authors for their response to all the reviewers' comments.

1) The authors responded to my comment about additional genomes by pointing out that they added a new assembly (peanut) and that data was not available for the other sequences due to funding and data retention reasons. I appreciate this response but maintain that these data being presented feels irrelevant with no genome or genome statistics presented as well.

2) With the addition of the peanut genome, the authors add some information on those timelines. In line 373, they comment that the peanut genome took nine additional days and provide a graphical timeline in the supplemental figures. I think an explanation of where the nine days are added (sequencing, assembly, data processing, etc) would be very helpful to have in the main body of the paper.

3) I appreciate that the authors added additional species for HMW DNA extraction in response to the concern of myself and other reviewers. However, each of the three species added to the study have been sequenced before. Do these methods work on species that have historically been very difficult to extract DNA from? Additionally, an improvement of sequence statistics between this method and previous ones could simply be attributed to the newer technologies used for sequencing.

4) I still do not feel that this paper presents a novel approach to genome assembly. Many labs are forgoing Illumina-based polishing for plant genomes. With near telomere-to-telomere contigs coming out of hifiasm, many are also forgoing Hi-C validation and other technologies as well.

Additional concerns I have:

5) Please provide a source for the claims that "The HiFi-based assemblies do not require additional polishing to the highly-accurate starting material." (line 517).

6) The authors claim in comments back to reviewer 1 (comment 5) that the ONT sequences were not suitable for a proper genome assembly. What evidence is there that the HMW extraction protocol works for ONT if the sequencing results could not produce an assembly?

7) Line 128: which eight species were sequenced with CLR? What happened to the other two?

8) Why is the ONT sequencing reported if it was not used for assembly?

While this paper is beautifully written, I still feel it is lacking as a methods paper. The main difference between this study and other published methods appears to be the repeated ethanol washes (line 412). The authors removed many mentions of "LeafGo" to simplify the paper for readers, but it feels even more ambiguous what the point of this paper is. I will repeat my sentiment from the prior review cycle that with additional analysis of the two eucalypt genomes, this could be a wonderful research paper in which they could still publish their method and modifications.

**Authors Response**

**Point-by-point responses to the reviewers’ comments:**

*We were happy to see that Reviewer 1 accepted our revisions without any further requests. However, as in the first review cycle, Reviewer 2 has repeated his comment that our manuscript is not novel. We acknowledge that each of the steps in our workflow are not novel by themselves. What we provide the research community is a novel resource: a complete workflow from sample collection, DNA extraction, long-read sequencing and data processing that yields high quality and contiguous genomes. Our workflow is ready to be used by anyone with the capability to do so. We provide guidance for sample collection, DNA extraction, library preparation and sequencing, and genome assembly.*

*Our DNA extraction protocol is thoroughly-tested, detailed and available online (dx.doi.org/10.17504/protocols.io.bafmibk6). We provide an abundance of data and statistics for all steps from DNA extraction and QC, library preparation, sequencing and data analysis. Furthermore, all the sequencing data used for our assemblies are submitted to public repositories. We also compare and benchmark current Pacbio sequencing technologies, as well as the current generation of appropriate genome assembly tools. In addition, despite the scientific community being aware of steps and methods for plant genome assembly, there is not a single published resource like ours, able to exploit the current breakthroughs and to generate high quality genomes at relatively low-cost, with limited resources and within days.*

*For example, researchers may be using a tried and tested DNA extraction method, sequencing technology or genome assembly tool that has not been optimized into a complete workflow. We are also confused by the fact that the apparent lack of novelty was not cause for Reviewer 2 to reject our manuscript during the first review cycle.*

*We understand the need to demonstrate the universality of our workflow to as many plant species as possible. Following the reviewers’ advice, we have included a further three species from three different families, including one species with a complex genome, peanut. We then assembled the sequencing data from the complex peanut genome, also as requested by the reviewers. However, with the resources available to us, we believe that we have demonstrated that our workflow is applicable to a broad range of plant species.*

*We thank Reviewer 2 for his/her constructive criticism, please see below for specific responses to his/her comments.*

Our point-by-point responses to Reviewer 2’s comments

1) The authors responded to my comment about additional genomes by pointing out that they added a new assembly (peanut) and that data was not available for the other sequences due to funding and data retention reasons. I appreciate this response but maintain that these data being presented feels irrelevant with no genome or genome statistics presented as well.

*We thank the reviewer for this comment and we might have failed in providing an exhaustive explanation. LeafGo is a complete workflow and we have presented data from the DNA extraction, sequencing, and assembly steps; however, considering the scope of our article we could not process all the species through to genome assembly. We extracted high molecular weight DNA from ten different species of plants. Eight out of ten of those species (80%) were sequenced, with different long-read technologies procedures, to evaluate the best laboratory practice for sequencing. We provided the sequencing statistics for all the sequenced species (Table S2), which are a key quality control to assess the sequencing and DNA extraction procedure. Those statistics and data are not irrelevant but are a valuable resource for scientists working in genomic centers. A subset of species (30%) was selected for assessment and benchmarking of the genome assembly component of our workflow. Three species (Pennisetum glaucum/pearl millet, Salicornia bigelovii/dwarf saltwort and Zea mays/sweetcorn), due to practical and funding constraints, have no PacBio HiFi data that we, and others, have shown is superior for genome assembly. A further two species (Distichlis palmeri/nipa grass and Salvadora persica/toothbrush tree) with sufficient HiFi data were not assembled for this present manuscript to allow for inclusion in future potential publications.*

2) With the addition of the peanut genome, the authors add some information on those timelines. In line 373, they comment that the peanut genome took nine additional days and provide a graphical timeline in the supplemental figures. I think an explanation of where the nine days are added (sequencing, assembly, data processing, etc) would be very helpful to have in the main body of the paper.

*We thank the reviewer for this suggestion. We would like to emphasize that all the time estimates we provide are approximations that the research community can use to assess our workflow; we supply all the information needed for the application of our workflow at other genomics centers with different sequencing and compute capabilities. When using our LeafGo workflow for the peanut, with a complex tetraploid 2.5 Gb genome, an additional nine days, compared to smaller genome eucalypts, are required to go from dark-treated leaves to an assembled genome. This additional time is the result of sequencing the larger genome to the coverage needed for assembly; we estimate approximately one day assembly/data processing (≤ 2 hours for eucalypts; 29.8 hours for peanut). This information is clearly presented in the Results (sub-section “Time estimates”; previous version lines: 286-293; revised version lines: 278-287). This information is shown graphically in Supplementary Figure S9 and precise computational requirements for the assembly of all the genomes are in Supplementary Table S5.*

*We have revised the part of the discussion (previous version line: 373; revised version lines: 367-368) mentioned: “The generation of the A. hypogaea genome took nine days longer than for a eucalypt species, due to the necessity of sequencing the larger genome at sufficient coverage with a consequential increase in platform sequencing time; however, significant reductions in sequencing time could be achieved by parallel sequencing on multiple machines.”*

3) I appreciate that the authors added additional species for HMW DNA extraction in response to the concern of myself and other reviewers. However, each of the three species added to the study have been sequenced before. Do these methods work on species that have historically been very difficult to extract DNA from? Additionally, an improvement of sequence statistics between this method and previous ones could simply be attributed to the newer technologies used for sequencing.

*We thank the reviewer for the comments. We do not think that newer sequencing technologies have affected the sequencing statistics in our work. Besides HiFi sequencing, which is new, we also included data from the well-established PacBio CLR and ONT long-read sequencing technologies.*

*The three species added were chosen following the suggestion of Reviewer 1 who required “evidence of high quality DNA isolation from Solanaceae, Leguminosae, or Cruciferae species” in order to be “represented enough for all plant kingdom”. The three new species used were the ones that were readily accessible to us in the timeframe available. We believe the prior sequencing of these species is a strength as comparisons can be made with our workflow. We disagree that our DNA extraction protocol has not been tested on difficult species. Data from two different Eucalyptus species, which are known to be challenging for DNA extraction (see Barbier et al. 2019, Plant Methods 15, 62, https://doi.org/10.1186/s13007-019-0447-3; Healey et al. 2014, Plant Methods 10, 21, https://doi.org/10.1186/1746-4811-10-21), due to the high polyphenolics and polysaccharide content, are presented in our manuscript. Anecdotally, from feedback to our preprint and online protocol, this method is being actively adopted by many plant scientists with other species.*

*Nonetheless, we appreciate that for the DNA extraction method in our workflow we only present data for ten species and many more species, including from other difficult species, could have been tested. Therefore, we are adding a sentence in the discussion (previous version line: 335; revised version lines: 327-329), “It should be highlighted that although we extracted high quality HMW DNA from ten different species, our extraction method might not work for all plant species.”*

4) I still do not feel that this paper presents a novel approach to genome assembly. Many labs are forgoing Illumina-based polishing for plant genomes. With near telomere-to-telomere contigs coming out of hifiasm, many are also forgoing Hi-C validation and other technologies as well.

*We agree with the reviewer on the importance of the recent developments in the field and it demonstrates the timeliness of our manuscript in providing supporting empirical evidence to the scientific community. For instance, the hifiasm method was only published five months ago (Cheng et al., 2021, Nature Methods 18, 170; https://doi.org/10.1038/s41592-020-01056-5) and the human telomere-to-telomere assemblies using HiFi data/mixed technologies are recently published (Miga et al., 2020, Nature, 585, 79, https://doi.org/10.1038/s41586-020-2547-7) or at the preprint stage (Nurk at al., 26th May 2021 https://doi.org/10.1101/2021.05.26.445798). For high-contiguity plant genomes, to date there are limited publications on HiFi sequencing and newer assembly methods (e.g. single plant genome using multiple technologies; preprint Wang et al., 8th June 2021 https://doi.org/10.1101/2021.06.08.447650; single plant genome preprint Sun et al., 17th May 2021 https://doi.org/10.1101/2021.05.15.444292), although these advances are becoming known in the field. This is exactly one of the goals of this manuscript: to provide a complete workflow using only one sequencing technology to generate chromosome assembly without other technologies. At present a hybrid approach is still commonly used (see above and e.g. preprint Wang et al., 4th January 2021 https://doi.org/10.1101/2021.01.03.424980). To help the development of a single sequencing technology approach, we compare and benchmark genome assembly using PacBio CLR and HiFi long-read sequencing. We also benchmarked current genome assembly tools, as originally requested by Reviewer 2 (Tables S4 and S5).*

*We reiterate that the novelty of our approach is in providing a complete workflow from plant tissue collection to genome assembly. None of the steps in our workflow are, in themselves, novel, despite our optimization and modifications, but combined into a complete cutting-edge pipeline that can be used off-the-shelf by many scientists in the plant community makes our manuscript novel.*

5) Please provide a source for the claims that "The HiFi-based assemblies do not require additional polishing to the highly-accurate starting material." (line 517).

*We agree that this sentence might be misleading and we have changed it as :*

*"The HiFi-based assemblies do not require additional polishing to the highly-accurate starting CCS sequences [42].", (previous version line: 517; revised version line: 512); Nurk et al 2020 (https://doi.org/10.1101/gr.263566.120).*

*We also added a supporting reference to a similar sentence in the Discussion: , “Although the eucalypt genomes assembled in this manuscript with both CLR and HiFi data are very contiguous, the higher base-level accuracy given by HiFi improves the assembly considerably thus removing the need for polishing with short-read sequencing [42].”, (previous version line: 338; revised version line: 332; Nurk et al 2020 (https://doi.org/10.1101/gr.263566.120))*

6) The authors claim in comments back to reviewer 1 (comment 5) that the ONT sequences were not suitable for a proper genome assembly. What evidence is there that the HMW extraction protocol works for ONT if the sequencing results could not produce an assembly?

and

8) Why is the ONT sequencing reported if it was not used for assembly?

*We agree with the reviewer and we have revised our manuscript (see below “Revised manuscript”).*

*We did not intend to claim that our ONT data was suitable for genome assembly. Our intention was to demonstrate the suitability of the HMW DNA extracted with our workflow for sequencing with ONT flow cells, (previous and revised version lines 102-104): “For the Eucalyptus species, libraries were produced and sequenced with the GridION platform demonstrating the suitability of the laboratory component of LeafGo for ONT sequencing.”; and (previous version lines: 163-164; revised version: Additional file: Oxford Nanopore Technology Sequencing, page 8): “Results demonstrated the suitability of our HMW extraction protocol for Oxford Nanopore sequencing (Figure S12 and Table S7)”.*

*For instance, some users of our workflow may only use the DNA extraction component for ONT sequencing. For these specific users, our ONT sequencing statistics will allow more informed assessment of our LeafGo workflow. Similar to our results for CLR and HiFi sequencing, we support our findings with the sequencing statistics reported in Table S7. The presented sequencing statistics (Yield, Number of Reads, percentage of Q7 bases, longest reads, Mean read length and N50) are the main parameters used to assess the sequencing quality (Fukasawa et al. 2020, G3, 4, 1193; https://doi.org/10.1534/g3.119.400864) of the extracted HMW DNA. In fact, the sequencing N50 (>30-40 Kb) we report exceeds many plant ONT studies except for really recent publications using specialized ultra-long library preparations (10th July 2021: Frei at al., 2021, GBE, evab159 https://doi.org/10.1093/gbe/evab159).*

*In response to the comments regarding the purpose of the ONT sequencing data and genome assembly, we would like to emphasize that our goal was not to use our ONT data for genome assembly as this is beyond the scope of this manuscript. In addition, in order to use our ONT data for genome assembly higher coverage would be needed by sequencing more flow cells, which is again, beyond the scope of our article. We completely agree with the reviewer that the main goal of the manuscript is presenting our LeafGo workflow, specifically using PacBio HiFi sequencing data for genome assembly, with relevant supporting evidence.*

*We have revised our manuscript to make it clearer. In order to avoid digression from the main focus of the manuscript, we moved the ONT sequencing results into the Supplementary Material.*

*Revised manuscript*

*Main text*

*Amended*

*Previous version lines: 102-104; revised version lines: 102-105: “For the Eucalyptus species, libraries were produced and sequenced with the GridION platform demonstrating the suitability of the laboratory component of LeafGo for ONT sequencing (Additional file 1: Oxford Nanopore Technology Sequencing).”*

*Additional file 1 (Supplementary Material)*

*Moved & Amended*

*Moved Main text, Results: “Oxford Nanopore Sequencing” (previous version lines: 162-170) into Supplementary Material, Additional File 1, Supplementary Results: Oxford Nanopore Technology Sequencing.*

*Amended text:*

*(previous version lines: 169-170): “Our results are in line with other ONT sequencing-based plant genomes [34, 40]” changed to (revised version, Additional File 1, Supplementary Results: Oxford Nanopore Technology Sequencing, page 8):*

*“Our sequencing results are in line with ONT sequencing of plant species, including eucalypts (Schalamun, et al., 2019, Mol. Ecol. Resour. 19, 77, https://doi.org/10.1111/1755-0998.12938; Choi, et al., 2020. Genome Biol. 21, 21, https://doi.org/10.1186/s13059-020-1938-2)”*

*Changed*

*Additional File 1: Supplementary Results: “Oxford Nanopore Technology Sequencing: effect of size selection method” changed to subsection “Effect of size selection method”*

*The ONT sequencing section was moved to the end of the supplementary results of the Additional file 1. To accommodate this change and to fit with the narration of the main manuscript, the layout of the Additional file 1 was reorganised as well as the order of supplementary tables and figures.*

7) Line 128: which eight species were sequenced with CLR? What happened to the other two?

*The full list of species sequenced and modality is reported in Tables S2 as indicated at line 130 (previous version). However, we agree with the reviewer that might not be clear so we add the names of the eight species sequenced (see below).*

*The “other two” refer to Brassica rapa (genome size 370 Mb) and Solanum melongena (genome size 1,210 Mb), two of the three added species which together with Arachis hypogaea (genome size 2,623 Mb) we used to further test our DNA extraction method as requested by Reviewer 1. Of those three species we sequenced with eight Sequel II HiFi SMRT cells, the one with the biggest genome size (Arachis hypogaea) reaching a total of eight different species sequenced with long reads technologies.*

*We have revised lines 128-132 of the previous version (revised version lines: 129-135) as follows, “The extracted HMW gDNA from eight of the species (Arachis hypogaea, Distichlis palmeri, Eucalyptus rudis, Eucalyptus. camaldulensis, Pennisetum glaucum, Salicornia bigelovii, Salvadora persica, and Zea mays), was processed into 26 CLR and 7 HiFi libraries prior to sequencing with the PacBio Sequel I (22: 1M SMRT cells) and Sequel II (17: 8M SMRT cells). SMRTLink analysis (additional file 1: Table S2) showed that sequence statistics for both CLR and HiFi libraries were above the PacBio recommended specifications [38] with optimal internal controls metrics indicating that no inhibition of the sequencing reaction was observed.”*

While this paper is beautifully written, I still feel it is lacking as a methods paper. The main difference between this study and other published methods appears to be the repeated ethanol washes (line 412). The authors removed many mentions of "LeafGo" to simplify the paper for readers, but it feels even more ambiguous what the point of this paper is. I will repeat my sentiment from the prior review cycle that with additional analysis of the two eucalypt genomes, this could be a wonderful research paper in which they could still publish their method and modifications.

*We thank the reviewer for his comments. We have revised our manuscript numerous times to increase the readability. We removed the numerous references to “LeafGo” in the manuscript on the excellent suggestion of the reviewers and also to improve the readability of the manuscript.*

*Regarding the format of the manuscript: following the first review cycle it was suggested that we reformat as a methods paper. We also think that our manuscript is best published as a methods article. We dispute that there is a previously published methods article covering the same topic and with the same scope as our manuscript. Our manuscript is not about DNA extraction alone - it is a complete method workflow from sample collection to genome assembly (as described above). We believe that this manuscript will make a very useful and important methods resource for the scientific community and a valuable addition to Genome Biology.*
